# Negligible Effect of Estrogen Deficiency on Development of Skeletal Changes Induced by Type 1 Diabetes in Experimental Rat Models

**DOI:** 10.1155/2020/2793804

**Published:** 2020-11-06

**Authors:** Aleksandra Janas, Ewa Kruczek, Piotr Londzin, Sławomir Borymski, Zenon P. Czuba, Joanna Folwarczna

**Affiliations:** ^1^Department of Pharmacology, Faculty of Pharmaceutical Sciences in Sosnowiec, Medical University of Silesia, Katowice, Poland; ^2^Institute of Biology, Biotechnology and Environmental Protection, Faculty of Natural Sciences, University of Silesia in Katowice, Poland; ^3^Department of Microbiology and Immunology, Faculty of Medical Sciences in Zabrze, Medical University of Silesia, Katowice, Poland

## Abstract

Although postmenopausal osteoporosis often occurs concurrently with diabetes, little is known about interactions between estrogen deficiency and hyperglycemia in the skeletal system. In the present study, the effects of estrogen deficiency on the development of biochemical, microstructural, and mechanical changes induced by streptozotocin-induced diabetes mellitus (DM) in the rat skeletal system were investigated. The experiments were carried out on nonovariectomized (NOVX) and ovariectomized (OVX) control and diabetic mature female Wistar rats. Serum levels of bone turnover markers (CTX-I and osteocalcin) and 23 cytokines, bone mass and mineralization, histomorphometric parameters, and mechanical properties of cancellous and compact bone were determined. The results were subjected to two-way ANOVA and principal component analysis (PCA). Estrogen deficiency induced osteoporotic changes, with increased bone resorption and formation, and worsening of microstructure (femoral metaphyseal BV/TV decreased by 13.0%) and mechanical properties of cancellous bone (the maximum load in the proximal tibial metaphysis decreased by 34.2%). DM in both the NOVX and OVX rats decreased bone mass, increased bone resorption and decreased bone formation, and worsened cancellous bone microarchitecture (for example, the femoral metaphyseal BV/TV decreased by 17.3% and 18.1%, respectively, in relation to the NOVX controls) and strength (the maximum load in the proximal tibial metaphysis decreased by 35.4% and 48.1%, respectively, in relation to the NOVX controls). Only in the diabetic rats, profound increases in some cytokine levels were noted. In conclusion, the changes induced by DM in female rats were only slightly intensified by estrogen deficiency. Despite similar effects on bone microstructure and strength, the influence of DM on the skeletal system was based on more profound systemic homeostasis changes than those induced by estrogen deficiency.

## 1. Introduction

Osteoporosis is a serious public health problem, leading, in developed countries, to fragility fractures in one of three women and in one of five men aged over 50 years [1]. Painful and disabling osteoporotic fractures not only worsen the quality of life but lead to premature death [2]. The main cause of osteoporosis is estrogen deficiency in postmenopausal women; however, other factors contribute to the development of the disease, including, among others, immobilization, administration of glucocorticosteroids, or diabetes [3].

There are two main types of diabetes: type 1 diabetes (T1D), with *β*-cell destruction and insulin deficiency, and type 2 diabetes (T2D), with progressive defect of insulin secretion on the background of insulin resistance [4]. Both types lead to numerous complications, including disorders of bone metabolism with increased fracture rate [5–7]. T1D, concerning about 5-10% of all diabetes cases, occurs in younger people, whereas T2D usually develops at a later age [4]. It is likely for T2D to occur concurrently with estrogen deficiency in postmenopausal women. However, increased life expectancy in T1D patients, resulting from the progress in the insulin therapy, increases the probability of its coexistence with postmenopausal osteoporosis.

It has been estimated that the trajectories of the epidemiology of diabetes and osteoporosis are similar, and the increase in the number of diabetic patients will contribute to the increase in the number of patients with osteoporosis induced by diabetes [8]. The clinical data on interactions between changes induced by estrogen deficiency and diabetes, concerning the skeletal system, are not abundant [9]. Also, little is known about interactions between estrogen deficiency and hyperglycemia in terms of effects on the skeletal system in experimental conditions *in vivo*.

Different experimental models are used in the preclinical bone pharmacology to investigate the effects of potential treatments on the development of osteoporotic changes. The well-established and almost fully adequate model of estrogen deficiency-induced postmenopausal osteoporosis is a model of bilaterally ovariectomized rats or mice [10]. The most widely used experimental model of T1D is diabetes induced by the administration of streptozotocin (STZ) in rats or mice (single *i.v*. or *i.p.* injection), which induces the destruction of pancreatic *β*-cells and severe hyperglycemia [11, 12]. As far as T2D is concerned, there are numerous different murine models used, none of them adequately mirroring the changes developing in the disease [13, 14].

The number of reports on the skeletal effects of diabetes in experimental models of estrogen deficiency is scarce. Up to very recently, there were early reports available, concerning mostly the effect on bone mineral density (BMD) and bone turnover markers [15–19]. However, in the last years, several reports have been published, shedding more light on the pathogenesis of changes developing under the influence of both pathologies in rodents [20–27].

The aim of the study was to evaluate the effects of estrogen deficiency on the development of biochemical, microstructural, and mechanical changes induced by T1D in the rat skeletal system. Taking into account the established role of some cytokines in the regulation of bone remodeling, we investigated the effects of estrogen deficiency and diabetes on the serum levels of a wide panel of cytokines, including both proinflammatory and anti-inflammatory ones.

## 2. Materials and Methods

### 2.1. Animals and Chemicals

Female Wistar rats were obtained from the Center of Experimental Medicine, Medical University of Silesia, Katowice, Poland. All procedures were approved by the Local Ethics Commission, Katowice, Poland (permission numbers: 9/2015 and 149/2015). The rats were maintained under monitored standard laboratory conditions according to the European Union guidelines (directive 2010/63/EU), in standard plastic cages (Tecniplast, Buguggiate, Italy), four-five rats per cage, under a 12 h light–12 h dark cycle (light on 7 : 00 a.m.). The experiment started after the acclimatization. The rats were 3 months old.

During the experiment, the following drugs were used: a drug used for the induction of T1D: streptozotocin (STZ; Cayman Chemical Company, Ann Arbor, MI, USA); drugs used in order to mark the calcification front: tetracycline hydrochloride (Sigma-Aldrich Co., St. Louis, MO, USA) and calcein (Sigma-Aldrich Co., St. Louis, MO, USA); drugs used for general anesthesia: ketamine (Bioketan; Vetoquinol Biowet Sp. z o.o., Gorzów Wielkopolski, Poland) and xylazine (Xylapan; Vetoquinol Biowet Sp. z o.o., Gorzów Wielkopolski, Poland). The rats were fed a standard laboratory diet (Labofeed B; Wytwórnia Pasz “Morawski”, Kcynia, Poland).

The experiments were performed on rats divided into the following groups (*n* = 9 − 10):

– Nonovariectomized control rats (NOVX)
– Ovariectomized control rats (OVX)
– Nonovariectomized rats with type 1 diabetes (DM-NOVX)
– Ovariectomized rats with type 1 diabetes (DM-OVX)

The initial body mass of the rats before the start of the procedures was similar. The bilateral ovariectomy was performed under ketamine-xylazine (*i.p.*) anesthesia. T1D was induced by a single dose of STZ (60 mg/kg *i.p.*, dissolved in 0.1 M citrate buffer), administered three days after the bilateral ovariectomy was performed. A week after the ovariectomy, the first measurement of nonfasting blood glucose level took place in order to confirm the development of diabetes, using an Accu-Chek Performa Nano glucometer (Roche Diagnostics GmbH, Mannheim, Germany) and Accu-Chek Performa test strips (Roche Diabetes Care, Mannheim, Germany). The blood samples for the measurement were taken from the tail vessels of conscious rats by cutting the tail tip. Rats with the blood glucose level above 400 mg/100 mL were regarded as diabetic. Nonfasting blood glucose level was then measured once a week. The rats were weighed once a week, and, furthermore, on the day before the end of the experiment. Moreover, since all the rats served as controls in a bigger study, they were subcutaneously administered saline (1 mL/kg/day), starting one week after the ovariectomy. In order to label the calcification front, tetracycline hydrochloride at a dose of 20 mg/kg *i.p.* was administered seven days after the ovariectomy, and then calcein (10 mg/kg *i.p.*) was administered three weeks after the administration of tetracycline hydrochloride.

The experiment was terminated five weeks after the ovariectomy. The rats were fasted, anesthetized with the *i.p.* administration of the mixture of ketamine and xylazine, and sacrificed by cardiac exsanguination. After the vital functions ceased, the liver, thymus, uterus, adrenal glands, and bones (right and left tibias, right and left femurs, L4 vertebra) were isolated. The soft organs and left bones and vertebras, after cleaning from soft tissues, were weighed on an Ohaus analytical balance model Adventurer Pro type AV264CM (Ohaus Europe GmbH, Greifensee, Switzerland). The length and diameter in the midlength of the left tibias and left femurs were measured using a digital caliper (VOREL 15240, Toya, Wrocław, Poland). The left femurs, left tibias, and the proximal part of the right femurs, cut in the middle of the bone length, were wrapped in gauze soaked with saline and stored below –20°C [28]. Blood serum was frozen at –80°C until subjected to further measurements.

### 2.2. Bone Mechanical Properties Studies

The mechanical properties of bones were evaluated with an Instron 3342 500N apparatus (Instron, Norwood, MA, USA). Bluehill 2 software (version 2.14; Instron, Norwood, MA, USA) was used for data analysis. Mechanical properties were assessed with the use of three-point bending tests in the left proximal tibial metaphysis and the left femoral diaphysis (test parameters: displacement rate of 0.01 mm/s, sampling rate of 100 Hz), as previously described [28–30].

To evaluate the strength of the proximal tibial metaphysis, the proximal epiphysis was removed, and the load was applied perpendicularly to the long axis of the bone 3 mm from its proximal edge [29, 30]. The following parameters were determined based on the load-displacement curves obtained for each bone: Young's modulus and the load, displacement, energy, and stress for the yield point (0.05% offset), maximum load point, and fracture point. In order to calculate the intrinsic (independent of bone size and shape) parameters: stress and Young's modulus, the tibial metaphysis was assumed to be a circular beam and its diameter measured with a digital caliper.

To determine the mechanical properties of the femoral diaphysis [28, 30], the load was applied perpendicularly to the diaphysis in the middle of the bone length, with the distance between the supporting points of 16 mm. The same parameters were determined as for the tibial metaphysis; the calculations were based on the assumption that the femoral diaphysis was an elliptical pipe. For calculations, the internal and external diameters of the right femoral diaphysis were measured in histological preparations of the transverse cross-section of the right femur in the midlength, with the use of the set consisting of a Carl Zeiss Axio Imager.A1 microscope (Carl Zeiss, Göttingen, Germany) connected with an Olympus DP71 camera (Olympus, Tokyo, Japan) and a computer with OsteoMeasure XP 1.3.0.1 software (OsteoMetrics Inc., Decatur, GA, USA), with a graphics tablet (model Cintiq 22HD; Wacom, Kazo, Japan).

The strength of the femoral neck was studied using a compression test [30]. The load was applied to the head of the femur along the long axis of the bone. The maximum load (inducing fracture) was measured.

### 2.3. Bone Composition and Mineralization Studies

After performing the mechanical tests, left tibias and femurs and L4 vertebras were lyophilized for ten days in FreeZone 6 lyophilizer (temperature: –51°C, pressure: 0.03 mBa; Labconco, Kansas City, MO, USA) and then mineralized at 640°C for 48 hours in a muffle furnace L9/11/C6 (Nabertherm, Lilienthal, Germany). The lyophilized and mineralized bones were weighed on an analytical balance Adventurer Pro type AV264CM (Ohaus Europe GmbH, Greifensee, Switzerland) in order to determine the mass of bone mineral, water, and organic substances in bones. The contents of bone mineral, water, and organic substances were calculated as the ratios to the bone mass. The ashed bones were dissolved in 6 M HCl and then diluted in distilled water to spectrophotometrically determine the calcium and phosphorus contents in the bone mineral, with the use of Pointe Scientific (Canton, MI, USA) kits.

### 2.4. Bone Histomorphometric Studies

The measurements of the transverse cross-sections of the tibial and femoral diaphysis were performed on undecalcified, unstained slides, prepared as previously described [30, 31]. The measurements of the longitudinal cross-sections of the femoral metaphysis were carried out on decalcified preparations which were stained with hematoxylin and eosin [32]. The OsteoMeasure system (OsteoMetrics Inc., Decatur, GA, USA) was used for the histomorphometric measurements, and the data were presented according to the American Society for Bone and Mineral Research (ASBMR) standardized nomenclature [33].

In the cancellous bone of the distal femoral metaphysis, the following parameters were determined: bone volume to tissue volume ratio (BV/TV), trabecular thickness (Tb.Th), trabecular separation (Tb.Sp), and trabecular number (Tb.N).

In cortical bone of the tibial and femoral diaphysis, the transverse cross-sectional area of the whole diaphysis (Tt.Ar), transverse cross-sectional area of the cortical bone (Ct.Ar), transverse cross-sectional area of the marrow cavity (Ma.Ar), and the Ma.Ar/Tt.Ar ratio were measured.

The periosteal and endosteal transverse growth (mineral apposition rate; MAR) in the tibial and femoral diaphysis were measured. However, the evaluation of the effect of diabetes on those parameters was impossible, because tetracycline and calcein labels could be observed only in the preparations of the nonovariectomized and ovariectomized controls.

### 2.5. Biochemical Studies

Serum concentrations of bone turnover markers, osteocalcin (a marker of bone formation) and C-terminal telopeptide fragments of type I collagen released from bone during bone resorption (CTX-I), were studied by the ELISA methods (Rat-MID Osteocalcin EIA and RatLaps (CTX-I) EIA, respectively, Immunodiagnostic Systems Ltd., Boldon, Tyne and Wear, UK). A microplate reader Stat Fax 2100 (Awareness Technology, Inc., Palm City, FL, USA) was used for the measurements.

Serum concentrations of calcium, inorganic phosphorus, glucose, fructosamine, and biochemical parameters of lipid metabolism (total cholesterol, high-density lipoprotein (HDL) cholesterol, low-density lipoprotein (LDL) cholesterol, and triglycerides) were determined spectrophotometrically, using kits produced by Pointe Scientific (Canton, MI, USA). Moreover, the concentration of advanced oxidation protein products (AOPP), a marker of oxidative protein damage, was measured spectrophotometrically based on the method of Witko-Sarsat et al. [34, 35]. The calibration curve was prepared using chloramine T (Sigma-Aldrich, St. Louis, MO, USA), and the absorbance was measured at the wavelength of 340 nm. The AOPP concentration was expressed in *μ*mol of chloramine T equivalents/L. The spectrophotometric measurements were performed with the use of Tecan Infinite M200 PRO microplate reader and Magellan 7.2 software (Tecan Austria GmbH, Grödig, Austria).

The measurements of serum concentrations of 23 cytokines were performed with the use of multiplex technology of magnetic bids combined with antibodies (Bio-Plex Pro Rat Cytokine 23-Plex Immunoassay; Bio-Rad Laboratories Inc., Hercules, CA, USA), using Bio-Plex 200 system and Bio-Plex Manager software (Bio-Rad). The full panel of 23 rat cytokines for which the technique was available was investigated. The following cytokines were assayed: interleukin-1*α* (IL-1*α*), IL-1*β*, IL-2, IL-4, IL-5, IL-6, IL-7, IL-10, IL-12p70, IL-13, IL-17A, IL-18, granulocyte colony-stimulating factor (G-CSF), granulocyte-macrophage colony-stimulating factor (GM-CSF), growth-regulated oncogene/keratinocyte chemoattractant (GRO/KC), interferon-*γ* (IFN-*γ*), macrophage colony-stimulating factor (M-CSF), macrophage inflammatory protein-1a (MIP-1a), macrophage inflammatory protein-3a (MIP-3a), regulated on activation, normal T cell expressed and secreted (RANTES), tumor necrosis factor *α* (TNF-*α*), vascular endothelial growth factor (VEGF), and monocyte chemoattractant protein-1 (MCP-1; monocyte chemotactic and activating factor—MCAF).

### 2.6. Statistical Analysis

Results are presented as the mean ± standard error of the mean (SEM). Statistical analysis was carried out with the use of two-way analysis of variance (ANOVA), with the main effects of estrogen deficiency (OVX) and type 1 diabetes (DM). In case of the statistical significance of any of the main effects or their interaction, the ANOVA was followed by Fisher's Least Significant Difference (LSD) *post hoc* test (Statistica 13.1; Tibco Software Inc., Palo Alto, CA, USA). *p* values < 0.05 were considered significant.

Moreover, the principal component analysis (PCA) was performed on the following groups of parameters:

– Body mass gain and mass of internal organs: the uterus, thymus, adrenals, and liver
– Serum metabolic biochemical parameters: concentrations of nonfasting and fasting blood glucose, triglycerides, total cholesterol, LDL cholesterol, HDL cholesterol, and AOPP
– Serum bone turnover parameters: CTX-I, osteocalcin, inorganic phosphorus, and total calcium concentrations
– Bone mass and macrometric parameters: tibial mass, length and diameter, femoral mass, length and diameter, and vertebral mass
– Bone composition and mineralization: the content of bone mineral, water, and organic substances in the tibia, femur, and vertebra (calculated as the ratios of their mass to the bone mass), content of calcium and phosphorus in the bone mineral in the tibia, femur, and vertebra
– Histomorphometric parameters of compact bone: Ma.Ar, Ct.Ar, Tt.Ar, and Ma.Ar/Tt.Ar in the tibial and femoral diaphysis
– Histomorphometric parameters of cancellous bone of the femoral metaphysis: BV/TV, BS/TV, BS/BV, Tb.Th, Tb.Sp, and Tb.N
– Mechanical properties of cancellous bone: Young's modulus, load, displacement, energy, and stress for yield point, maximum load point, and fracture point in the tibial metaphysis
– Mechanical properties of compact bone: Young's modulus, load, displacement, energy, and stress for yield point, maximum load point, and fracture point in the femoral diaphysis
– Serum concentrations of 23 cytokines

The PCA was carried out using the PAST 3.26 software [36, 37]. Correlation matrices were employed for data standardization. The correlation values presented on the graphs are autoscaled based on the algorithm built into the software. Statistical significance of the PCA results was evaluated using two-way MANOVA (multivariate analysis of variance; Statistica 13.1). In case of the statistical significance of any of the main effects or their interaction, further analysis for individual axes (PC1, PC2) was performed, followed by Fisher's LSD *post hoc* test. Only significant *post hoc* differences between DM-OVX versus DM-NOVX are mentioned in the figure legends; all results—see Supplementary Material 1.

## 3. Results

### 3.1. General Parameters

At the end of the experiment, five weeks after the bilateral ovariectomy surgery, the mean body mass of the diabetic rats, both nonovariectomized and ovariectomized, significantly decreased in relation to appropriate control rats, whereas the mean body mass of the nondiabetic ovariectomized rats significantly increased (Table 1).

The liver mass increased, and the mass of the uterus, thymus, and adrenal glands decreased in the diabetic rats independent of the estrogen status, whereas the uterus mass decreased, and thymus mass increased in the nondiabetic ovariectomized rats.

Both the nonovariectomized and ovariectomized rats administered STZ developed diabetes, as indicated by profoundly increased glucose and fructosamine levels in relation to appropriate controls, moreover, significant disorders of lipid metabolism were noted (Table 2). Estrogen deficiency itself did not affect the parameters connected with glucose metabolism; however, it increased the cholesterol and LDL cholesterol levels. The estrogen-deficient diabetic rats had significantly increased the level of AOPP in relation to the ovariectomized controls, indicating oxidative protein damage.

**Figure 1:**
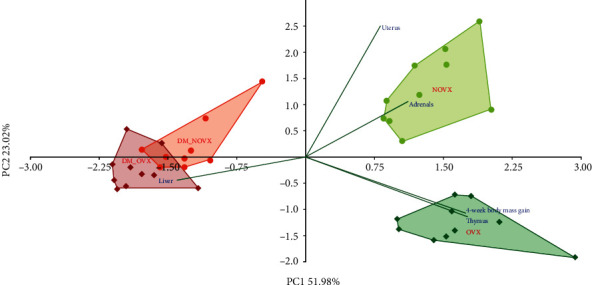
PCA biplot of the results concerning the body mass gain and the mass of internal organs (uterus, thymus, adrenal glands, and liver) in the diabetic and/or estrogen-deficient rats. Bilateral ovariectomy was performed 5 weeks before the end of the experiment. Diabetes was induced 3 days after the ovariectomy by a single administration of streptozotocin (60 mg/kg *i.p.*). Blue font and green lines indicate correlations of measured variables against experimental groups. Shades of red denote DM rats; shades of green denote nondiabetic rats. Darker shades and diamond symbols denote OVX rats; lighter shades and dot symbols denote NOVX rats. Group names are marked in red fonts inside the convex hulls comprised by individuals from the same group. The statistical significance between the groups along the PCs was analysed by two-way MANOVA. Significant results for PC1: DM—*p* < 0.001; OVXxDM interaction—*p* < 0.01; and for PC2: OVX—*p* < 0.001, OVXxDM interaction—*p* < 0.001. *Post hoc* LSD for PC1: DM-NOVX versus DM-OVX—*p* < 0.01. Detailed data—see Supplementary Material 1A.

**Figure 2:**
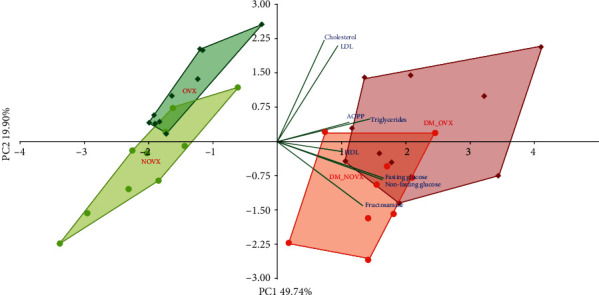
PCA biplot of the results concerning the concentrations of the serum biochemical metabolic parameters in the diabetic and/or estrogen-deficient rats. Bilateral ovariectomy was performed 5 weeks before the end of the experiment. Diabetes was induced 3 days after the ovariectomy by a single administration of streptozotocin (60 mg/kg *i.p.*). Blue font and green lines indicate correlations of measured variables against experimental groups. Shades of red denote DM rats; shades of green denote nondiabetic rats. Darker shades and diamond symbols denote OVX rats; lighter shades and dot symbols denote NOVX rats. Group names are marked in red fonts inside the convex hulls comprised by individuals from the same group. The statistical significance between the groups along the PCs was analysed by two-way MANOVA. Significant results for PC1: OVX—*p* < 0.05; DM—*p* < 0.001, and for PC2: OVX—*p* < 0.001; DM—*p* < 0.05. *Post hoc* LSD for PC2: DM-NOVX versus DM-OVX—*p* < 0.01. Detailed data—see Supplementary Material 1B.

The PCA indicated strong differences concerning the body mass gain, internal organ mass, and serum biochemical metabolic parameters between the diabetic and nondiabetic rats, as well as the nonovariectomized and ovariectomized rats (Figures 1 and 2, respectively).

### 3.2. Serum Bone Turnover Markers

**Figure 3:**
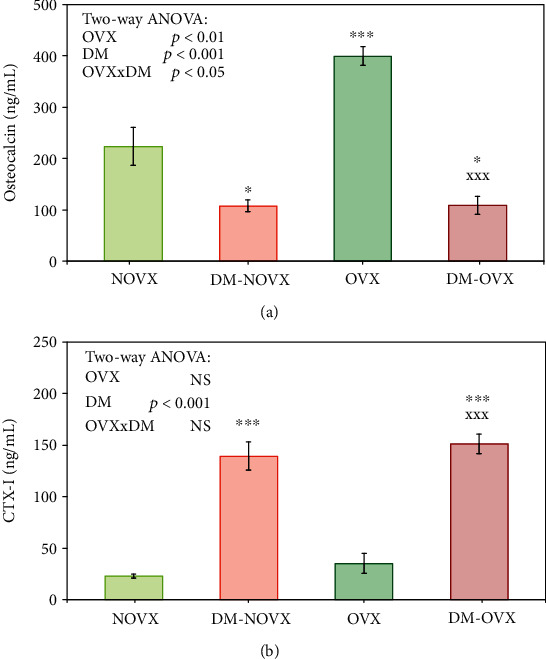
Effects of diabetes (DM) and/or estrogen deficiency on the serum bone turnover markers (osteocalcin and CTX-I) in rats. Bilateral ovariectomy was performed 5 weeks before the end of the experiment. Diabetes was induced 3 days after the ovariectomy by a single administration of streptozotocin (60 mg/kg *i.p.*). Results are presented as means ± SEM (*n* = 9 − 10). NOVX: nonovariectomized control rats; DM-NOVX: rats with type 1 diabetes; OVX: ovariectomized control rats; DM-OVX: ovariectomized rats with type 1 diabetes. CTX-I: C-terminal telopeptide fragments of type I collagen. Two-way ANOVA followed by Fisher's LSD test was used for evaluation of the significance of the results. ^∗^*p* < 0.05, ^∗∗∗^*p* < 0.001—significantly different from the NOVX control rats; ^XXX^*p* < 0.001—significantly different from the OVX control rats.

Diabetes induced significant increases in the concentration of the bone resorption marker CTX-I (by 503.9% in the nonovariectomized rats and by 325.9% in the ovariectomized rats in relation to the appropriate nondiabetic controls) and decreases in that of the bone formation marker osteocalcin (by 51.5% in the nonovariectomized rats and by 72.7% in the ovariectomized rats in relation to the appropriate nondiabetic controls) (Figure 3). Estrogen deficiency induced a significant increase in the osteocalcin concentration only in the nondiabetic rats (by 78.7% in relation to the nonovariectomized controls); the CTX-I level was insignificantly increased (by 54.0% in relation to the nonovariectomized controls). Diabetes induced increases in the calcium concentrations and decreases in the inorganic phosphorus concentrations, regardless of the estrogen status (Table 2).

**Figure 4:**
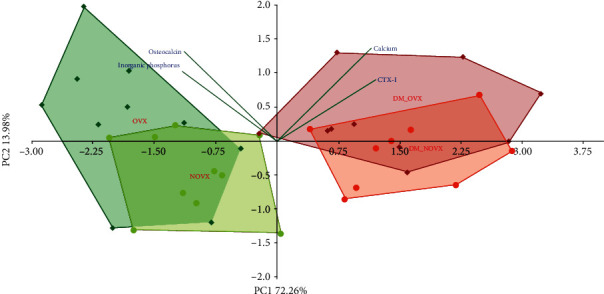
PCA biplot of the results concerning the concentrations of the serum bone turnover markers and concentrations of calcium and inorganic phosphorus in the diabetic and/or estrogen-deficient rats. Bilateral ovariectomy was performed 5 weeks before the end of the experiment. Diabetes was induced 3 days after the ovariectomy by a single administration of streptozotocin (60 mg/kg *i.p.*). Blue font and green lines indicate correlations of measured variables against experimental groups. Shades of red denote DM rats; shades of green denote nondiabetic rats. Darker shades and diamond symbols denote OVX rats; lighter shades and dot symbols denote NOVX rats. Group names are marked in red fonts inside the convex hulls comprised by individuals from the same group. The statistical significance between the groups along the PCs was analysed by two-way MANOVA. Significant results for PC1: DM—*p* < 0.001, and for PC2: OVX—*p* < 0.01. Detailed data—see Supplementary Material 1C.

The PCA analysis indicated strong differences concerning the serum concentrations of bone turnover markers, calcium, and inorganic phosphorus between the diabetic and nondiabetic rats, as well as the nonovariectomized and ovariectomized rats (Figure 4).

### 3.3. Bone Mass, Macrometric Parameters, Composition, and Mineralization

Regardless the estrogen status, the diabetic rats had significantly decreased the bone mass, bone mineral mass, and bone mineral content (the bone mineral mass/bone mass ratio) both in the long bones and the vertebra, in relation to appropriate controls (Table 3, data for the tibia not shown). For example, the bone mineral content in the femur of the nonovariectomized diabetic rats decreased by 4.1% in comparison with the nonovariectomized controls, and that of the ovariectomized diabetic rats—by 3.2% in comparison with the ovariectomized controls. Also, the femoral length decreased. There was no effect of diabetes on the bone content of water and organic substances, as well as on the calcium content in the bone mineral. Estrogen deficiency did not significantly affect the abovementioned parameters, with the exception of decreasing the bone mineral content (by 2.6%) concomitantly with increasing the bone water content in the femur in relation to the nonovariectomized controls.

**Figure 5:**
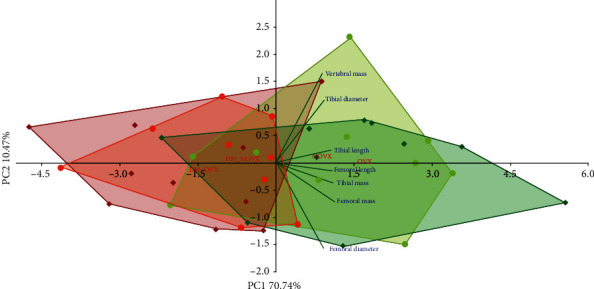
PCA biplot of the results concerning the bone mass and macrometric parameters in the diabetic and/or estrogen-deficient rats. Bilateral ovariectomy was performed 5 weeks before the end of the experiment. Diabetes was induced 3 days after the ovariectomy by a single administration of streptozotocin (60 mg/kg *i.p.*). Blue font and green lines indicate correlations of measured variables against experimental groups. Shades of red denote DM rats; shades of green denote nondiabetic rats. Darker shades and diamond symbols denote OVX rats; lighter shades and dot symbols denote NOVX rats. Group names are marked in red fonts inside the convex hulls comprised by individuals from the same group. The statistical significance between the groups along the PCs was analysed by two-way MANOVA. Significant results for PC1: DM—*p* < 0.001. Detailed data—see Supplementary Material 1D.

**Figure 6:**
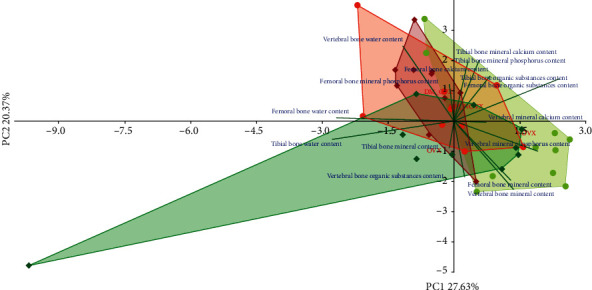
PCA biplot of the results concerning the bone composition and mineralization in the diabetic and/or estrogen-deficient rats. Bilateral ovariectomy was performed 5 weeks before the end of the experiment. Diabetes was induced 3 days after the ovariectomy by a single administration of streptozotocin (60 mg/kg *i.p.*). Blue font and green lines indicate correlations of measured variables against experimental groups. Shades of red denote DM rats; shades of green denote nondiabetic rats. Darker shades and diamond symbols denote OVX rats; lighter shades and dot symbols denote NOVX rats. Group names are marked in red fonts inside the convex hulls comprised by individuals from the same group. The statistical significance between the groups along the PCs was analysed by two-way MANOVA. Significant results for PC2: DM—*p* < 0.01. Detailed data—see Supplementary Material 1E.

The PCA analysis indicated differences concerning the bone mass and macrometric parameters (Figure 5) and bone composition and mineralization (Figure 6) between the diabetic and nondiabetic rats.

### 3.4. Bone Histomorphometry

**Figure 7:**
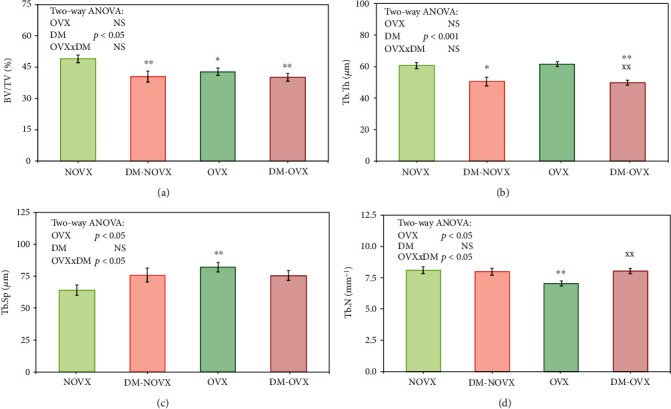
Effects of diabetes (DM) and/or estrogen deficiency on the histomorphometric parameters of cancellous bone of the distal femoral metaphysis in rats. Bilateral ovariectomy was performed 5 weeks before the end of the experiment. Diabetes was induced 3 days after the ovariectomy by a single administration of streptozotocin (60 mg/kg *i.p.*). Results are presented as means ± SEM (*n* = 9 − 10). NOVX: nonovariectomized control rats; DM-NOVX: rats with type 1 diabetes; OVX: ovariectomized control rats; DM-OVX: ovariectomized rats with type 1 diabetes. BV/TV: bone volume to tissue volume ratio; Tb.Th: trabecular thickness; Tb.Sp: trabecular separation; Tb.N: trabecular number. Two-way ANOVA followed by Fisher's LSD test was used for evaluation of the significance of the results. ^∗^*p* < 0.05, ^∗∗^*p* < 0.01—significantly different from the NOVX control rats; ^XX^*p* < 0.01—significantly different from the OVX control rats.

Diabetes induced deterioration of microarchitecture of the cancellous bone of the femoral metaphysis, regardless of the estrogen status (Figure 7). In the nonovariectomized rats, diabetes decreased the bone volume to tissue volume ratio (BV/TV) and trabecular thickness (Tb.Th), whereas in the ovariectomized rats—Tb.Th was decreased and trabecular number (Tb.N) was increased due to diabetes. Estrogen deficiency, in the nondiabetic rats, induced significant decreases in BV/TV and Tb.N, as well as increases in trabecular separation (Tb.Sp). The changes in BV/TV induced by diabetes, estrogen deficiency, or both pathologies were of similar degree (decreases by 17.3%, 13.0%, and 18.1%, respectively, in relation to the nonovariectomized controls).

In cortical bone, diabetes decreased the transverse cross-sectional area of the cortical bone (Ct.Ar) and transverse cross-sectional area of the whole diaphysis (Tt.Ar), both in the tibial and femoral diaphysis, regardless the estrogen status (Table 4, data for the tibial diaphysis not shown). The Ct.Ar decreased, due to diabetes, by 5.8% in the nonovariectomized rats, and by 9.0% in the ovariectomized rats, in relation to the respective control rats. Estrogen deficiency did not affect the histomorphometric parameters of cortical bone.

**Figure 8:**
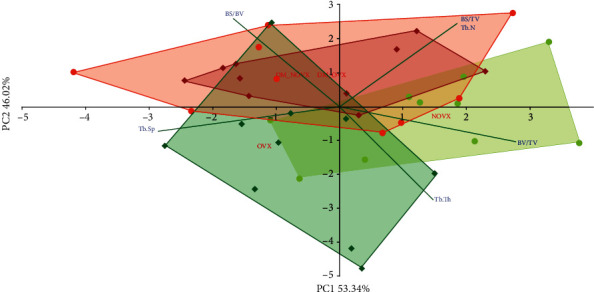
PCA biplot of the results concerning histomorphometric parameters of cancellous bone (the distal femoral metaphysis) in the diabetic and/or estrogen-deficient rats. Bilateral ovariectomy was performed 5 weeks before the end of the experiment. Diabetes was induced 3 days after the ovariectomy by a single administration of streptozotocin (60 mg/kg *i.p.*). Blue font and green lines indicate correlations of measured variables against experimental groups. Shades of red denote DM rats; shades of green denote nondiabetic rats. Darker shades and diamond symbols denote OVX rats; lighter shades and dot symbols denote NOVX rats. Group names are marked in red fonts inside the convex hulls comprised by individuals from the same group. The statistical significance between the groups along the PCs was analysed by two-way MANOVA. Significant results for PC2: DM—*p* < 0.001. Detailed data—see Supplementary Material 1F.

**Figure 9:**
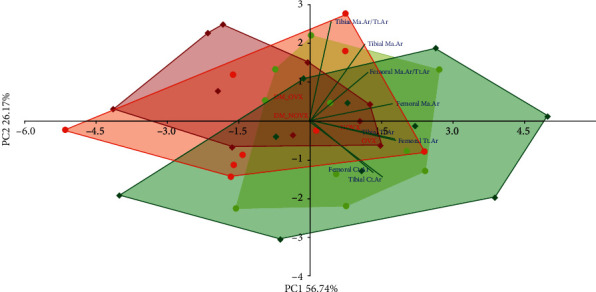
PCA biplot of the results concerning histomorphometric parameters of compact bone (the tibial and femoral diaphysis) in the diabetic and/or estrogen-deficient rats. Bilateral ovariectomy was performed 5 weeks before the end of the experiment. Diabetes was induced 3 days after the ovariectomy by a single administration of streptozotocin (60 mg/kg *i.p.*). Blue font and green lines indicate correlations of measured variables against experimental groups. Shades of red denote DM rats; shades of green denote nondiabetic rats. Darker shades and diamond symbols denote OVX rats; lighter shades and dot symbols denote NOVX rats. Group names are marked in red fonts inside the convex hulls comprised by individuals from the same group. The statistical significance between the groups along the PCs was analysed by two-way MANOVA. Significant results for PC1: DM—*p* < 0.05. Detailed data—see Supplementary Material 1G.

The PCA analysis indicated differences concerning histomorphometric parameters of cancellous (Figure 8) and compact (Figure 9) between the diabetic and nondiabetic rats.

### 3.5. Bone Mechanical Properties

**Figure 10:**
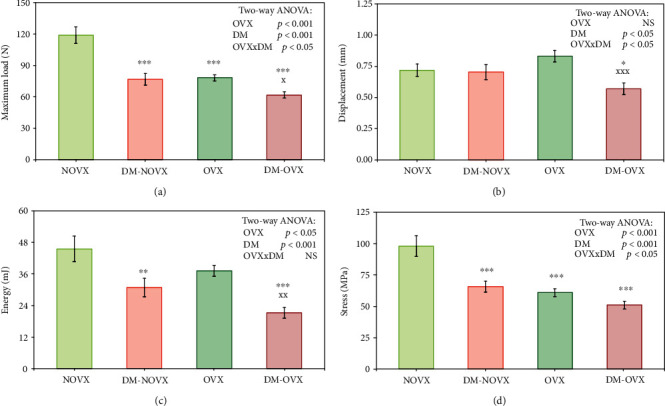
Effects of diabetes (DM) and/or estrogen deficiency on the mechanical properties of cancellous bone (the proximal tibial metaphysis – data for the maximum load point) in rats. Bilateral ovariectomy was performed 5 weeks before the end of the experiment. Diabetes was induced 3 days after the ovariectomy by a single administration of streptozotocin (60 mg/kg *i.p.*). Results are presented as means ± SEM (*n* = 9 − 10). NOVX: nonovariectomized control rats; DM-NOVX: rats with type 1 diabetes; OVX: ovariectomized control rats; DM-OVX: ovariectomized rats with type 1 diabetes. Two-way ANOVA followed by Fisher's LSD test was used for evaluation of the significance of the results. ^∗^*p* < 0.05, ^∗∗^*p* < 0.01, ^∗∗∗^*p* < 0.001—significantly different from the NOVX control rats; ^X^*p* < 0.05, ^XX^*p* < 0.01, ^XXX^*p* < 0.001—significantly different from the OVX control rats.

In the proximal tibial metaphysis, consisting mostly of cancellous bone, diabetes induced worsening of bone strength, regardless of the estrogen status (Figure 10, Table 5; data for the yield point not shown). In the ovariectomized control rats, most of the mechanical parameters were similar to those observed in the nonovariectomized diabetic rats. Moreover, in the ovariectomized control rats, Young's modulus was significantly decreased in relation to the nonovariectomized controls. The changes in the ovariectomized diabetic rats were in most cases less pronounced than in the nonovariectomized diabetic rats in relation to the appropriate nondiabetic controls, as indicated by significant interactions concerning the mechanical parameters of the tibial metaphysis noted in two-way ANOVA. For example, the decreases in the maximum load induced by diabetes, estrogen deficiency, or both pathologies were as follows: 35.4%, 34.2%, and 48.1%, respectively, in relation to the nonovariectomized control rats. However, the maximum load in the ovariectomized diabetic rats was decreased only by 21.1% in comparison with the ovariectomized controls.

In the femoral diaphysis, no effects of diabetes on the extrinsic mechanical parameters were observed; however, increases in the intrinsic parameters (Young's modulus in both the nonovariectomized and ovariectomized rats, stress in the nonovariectomized rats) were observed (Table 5; data for the yield point not shown). The value of Young's modulus increased due to diabetes by 21.9% in the nonovariectomized rats and by 11.1% in the ovariectomized rats in comparison with appropriate controls. Estrogen deficiency alone did not affect the investigated parameters, whereas in the diabetic rats, it decreased the value of stress in relation to the nonovariectomized diabetic rats.

There was no effect of both diabetes and estrogen deficiency on the strength of the femoral neck (not shown).

**Figure 11:**
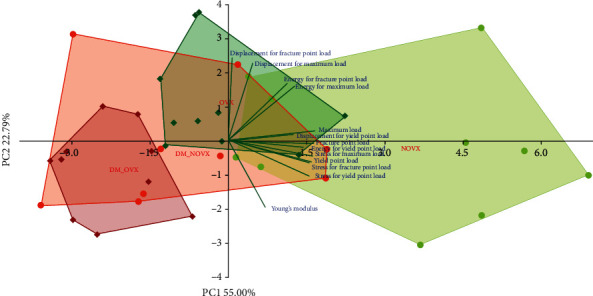
PCA biplot of the results concerning mechanical properties of cancellous bone (the proximal tibial metaphysis) in the diabetic and/or estrogen-deficient rats. Bilateral ovariectomy was performed 5 weeks before the end of the experiment. Diabetes was induced 3 days after the ovariectomy by a single administration of streptozotocin (60 mg/kg *i.p.*). Blue font and green lines indicate correlations of measured variables against experimental groups. Shades of red denote DM rats; shades of green denote nondiabetic rats. Darker shades and diamond symbols denote OVX rats; lighter shades and dot symbols denote NOVX rats. Group names are marked in red fonts inside the convex hulls comprised by individuals from the same group. The statistical significance between the groups along the PCs was analysed by two-way MANOVA. Significant results for PC1: OVX—*p* < 0.001; DM—*p* < 0.001. Detailed data—see Supplementary Material 1H.

**Figure 12:**
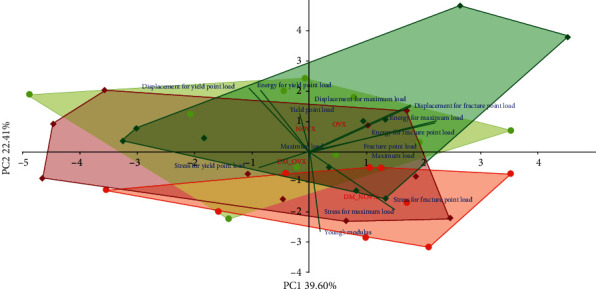
PCA biplot of the results concerning mechanical properties of compact bone (the femoral diaphysis) in the diabetic and/or estrogen-deficient rats. Bilateral ovariectomy was performed 5 weeks before the end of the experiment. Diabetes was induced 3 days after the ovariectomy by a single administration of streptozotocin (60 mg/kg *i.p.*). Blue font and green lines indicate correlations of measured variables against experimental groups. Shades of red denote DM rats; shades of green denote nondiabetic rats. Darker shades and diamond symbols denote OVX rats; lighter shades and dot symbols denote NOVX rats. Group names are marked in red fonts inside the convex hulls comprised by individuals from the same group. The statistical significance between the groups along the PCs was analysed by two-way MANOVA. Significant results for PC2: DM—*p* < 0.01. Detailed data—see Supplementary Material 1I.

The PCA analysis indicated differences concerning the mechanical properties of cancellous (Figure 11) and compact bone (Figure 12) between the diabetic and nondiabetic rats. Moreover, there were differences concerning the mechanical properties of cancellous bone between the nonovariectomized and ovariectomized rats.

### 3.6. Serum Cytokine Concentrations

STZ-induced diabetes led to the development of profound changes in the serum cytokine levels, both in the nonovariectomized and ovariectomized rats (significant main diabetes effects) (Table 6). The following cytokines were moderately (less than 5x) increased: IL-1*α*, IL-4, IL-10, IL-13, IL-17A, G-CSF, IFN-*γ*, M-CSF, MIP-3a, RANTES, and TNF-*α*, and the following were strongly increased (more than 5x): IL-1*β*, IL-7, GM-CSF, GRO/KC, MIP-1a, VEGF, and MCP-1 (MCAF). There were no significant changes in the cytokine levels induced by estrogen deficiency. The only significant interaction between the main effects (OVX and DM) concerned the RANTES concentration, which was increased only in the diabetic ovariectomized rats.

**Figure 13:**
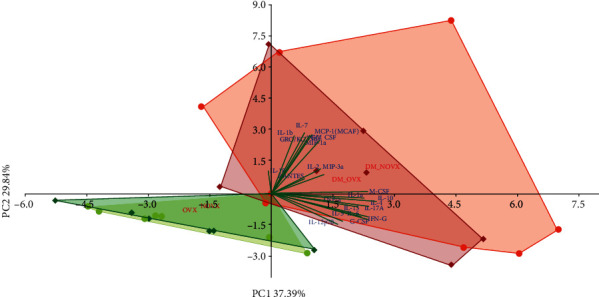
PCA biplot of the results concerning the serum concentrations of 23 cytokines in the diabetic and/or estrogen deficiency rats. Bilateral ovariectomy was performed 5 weeks before the end of the experiment. Diabetes was induced 3 days after the ovariectomy by a single administration of streptozotocin (60 mg/kg *i.p.*). Blue font and green lines indicate correlations of measured variables against experimental groups. Shades of red denote DM rats; shades of green denote nondiabetic rats. Darker shades and diamond symbols denote OVX rats; lighter shades and dot symbols denote NOVX rats. Group names are marked in red fonts inside the convex hulls comprised by individuals from the same group. The statistical significance between the groups along the PCs was analysed by two-way MANOVA. Significant results for PC1: DM—*p* < 0.001, and for PC2: DM—*p* < 0.05. Detailed data—see Supplementary Material 1J.

The PCA analysis indicated strong differences concerning cytokine levels between the diabetic and nondiabetic rats, regardless of the estrogen status (Figure 13).

## 4. Discussion

Although postmenopausal osteoporosis often occurs concurrently with diabetes, little is known about interactions between estrogen deficiency and hyperglycemia in terms of effects on the skeletal system *in vivo*.

In the present study, bilateral ovariectomy induced estrogen deficiency, as confirmed by characteristic changes in estrogen-dependent organ mass (the uterus and thymus), significant increases in the body mass gain, and serum cholesterol levels. Also, osteoporotic microstructure and strength changes due to estrogen deficiency were demonstrated five weeks after the ovariectomy, as previously described [30, 38]. The microstructure changes concerned cancellous bone (decreases in BV/TV and Tb.N, increases in Tb.Sp) and led to worsening of the strength of the proximal tibial metaphysis, built mostly of cancellous bone. There were no significant changes in compact bone histomorphometric parameters and mechanical properties (Ct.Ar, Tt.Ar, Ma.Ar in the tibial and femoral diaphysis, and strength of the femoral diaphysis, respectively). The bone resorption marker (CTX-I) concentration tended to increase, and the bone formation marker (osteocalcin) level significantly increased. The observed changes are consistent with well-established mechanisms of changes developing in the bone due to estrogen deficiency. Estrogen deficiency increases the rate of bone remodeling, and both bone resorption and formation accelerate. This is because estrogen decreases the number of remodeling cycles, inhibiting the birth rate of osteoclasts and osteoblasts from their progenitors, and has proapoptotic effects on osteoclasts and antiapoptotic effects on osteoblasts and osteocytes. The loss of bone mass and strength is the result of the shift of the balance toward bone resorption [39].

The nonovariectomized rats administered STZ developed diabetes, with hyperglycemia, polydipsia, polyuria, characteristic changes in the serum parameters, and decreased body mass. Very profound changes in the skeletal system were observed, consistent with other studies [18, 20–25]. Bone mass, mineralization, and mechanical properties of cancellous bone of the proximal tibial metaphysis profoundly worsened, whereas the strength of compact bone was not decreased. In fact, significant increases in the values of intrinsic mechanical parameters (stress, Young's modulus) in the femoral diaphysis were demonstrated in the diabetic rats, which however was rather the result of decreases in bone size than improvement of bone quality. Deterioration of cancellous bone microstructure was demonstrated (decreases in BV/TV and Tb.Th). Bone resorption was intensified, and bone formation was decreased as demonstrated by the measurements of serum bone turnover marker concentrations (CTX-I and osteocalcin, respectively).

The pathomechanism of the development of the skeletal changes in diabetes is complex. There are differences between clinical skeletal manifestations of type 1 and type 2 diabetes. T1D leads to decreased BMD and increased fracture risk. In T2D, the decreases in BMD do not occur; however, the risk of fracture is also elevated [5, 6, 40]. In T1D, hyperglycemia and the inflammatory environment influence all types of bone cells, i.e., osteoblasts, osteocytes, and osteoclasts [41]. The mechanisms of the development of the bone changes, demonstrated in experimental conditions, involved, among others: intensification of osteoclast activity, decreased function of osteoblasts induced by insulin deficiency, and increased expression of sclerostin by osteocytes, as well as decreased bone quality caused by advanced glycation products [41, 42]. Moreover, diabetes increases oxidative stress [43, 44], which may intensify bone resorption and inhibit bone formation [39].

Summing up, the ovariectomy and STZ administration induced opposite actions on the body mass gain. Bone mass and macrometric parameters were not affected by estrogen deficiency, whereas diabetes induced significant decreases in the bone mass, length, and femoral diaphysis cross-section area. Nonetheless, the bone quality changes in the diabetic rats in the present study were very similar to those developing due to estrogen deficiency. In both experimental models, cancellous bone mechanical properties were worsened to almost the same extent, and there was no damaging effect on the strength of the compact bone of the femoral diaphysis. The differences concerning cancellous bone microstructure were slight: the decrease in BV/TV was caused by a decrease in Tb.N with an increase in Tb.Sp in the ovariectomized control rats, whereas in the diabetic rats, by a decrease in Tb.Th. The main difference between the models concerned the bone turnover markers. Although both endocrine changes induced increases in bone resorption, estrogen deficiency increased bone formation marker concentration, whereas diabetes decreased it. The decrease in bone formation may explain the decrease in Tb.Th in the diabetic rats.

The skeletal effects of diabetes in the estrogen-deficient rats were very similar to those induced in the rats with normal estrogen levels (nonovariectomized). No further decrease in BV/TV was demonstrated, and Tb.Th decreased and Tb.N increased in relation to the ovariectomized control rats. However, further deterioration in the mechanical properties of cancellous bone compared to the estrogen-deficient control rats was observed. Similarly to the effects in the nonovariectomized rats, diabetes induced an increase in Young's modulus of the femoral diaphysis (compact bone) of the ovariectomized rats; however, no increases in the stress values were demonstrated. The bone resorption marker (CTX-I) further increased, and bone formation marker (osteocalcin) strongly decreased in relation to both the ovariectomized and nonovariectomized control rats. These observations are partially at variance with those of Liu et al. [25] who concluded, based on microstructural and gene expression studies, that STZ-induced diabetes, in estrogen-deficient rats, reversed high bone turnover osteoporosis into low bone turnover one (decreases in both formation and bone resorption indices), with stronger microstructure changes and bone loss. On the other hand, Wen et al. [20] reported increasing effects of concurrent diabetes and estrogen deficiency on the rat serum bone turnover markers and osteoblast and osteoclast number in bone tissue, similarly to the effects induced by estrogen deficiency alone (whereas in diabetes alone, bone formation indices were decreased). The mouse studies indicated possible roles of renin-angiotensin and kallikrein-kinin systems [24] and increased expression of TNF-*α* [23] in the development of osteoporotic changes induced by diabetes and estrogen deficiency.

There are few studies that investigated bone mechanical properties in diabetic osteoporotic animals. As far as mechanical properties of cortical bone are concerned, de Mello-Sampayo et al. [22] reported on the favorable effect of hyperglycemia on the femoral strength, since the values of ultimate stress and Young's modulus increased in estrogen-deficient diabetic rats. We also observed increases in Young's modulus values for the femoral diaphysis of the diabetic rats, regardless of the estrogen status. Also, the maximum stress increased, but only in the nonovariectomized diabetic rats. The lack of such an effect in the estrogen-deficient rats does not support the hypothesis on the favorable effects of diabetes (hyperglycemia) on the mechanical properties of cortical bone. Moreover, it should be stressed that diabetes induced strong harmful effect on the mechanical properties of the cancellous bone of the tibial metaphysis, both in the nonovariectomized and ovariectomized rats. Similarly to our results for cancellous bone, no significant differences between the L4 vertebra strength of nonovariectomized and ovariectomized diabetic rats were observed by Lee et al. [21] (the effects of estrogen deficiency alone were not investigated in that study).

The differences between the results concerning the effects of diabetes and estrogen deficiency on the skeletal system in different studies might have resulted from differences in the study designs. Stronger than in the present study aggravation of damaging effects of both pathologies over those induced by them separately were reported by other authors in mouse models [23, 24] or after longer periods of observation in rats [20, 21, 25]. However, the measurements were performed five weeks after the ovariectomy in the present study. Such a period in adult rats corresponds to about three years in humans [45]. The period of four-five weeks was long enough to demonstrate the effects of different treatments or estrogen deficiency on the skeletal system in rats in our previous studies [30, 38].

There are interactions between estrogen deficiency and energy metabolism. For example, it has been reported that long-term estrogen deficiency leads to the decline of whole-body glucose metabolism in ovariectomized rats [46]. However, based on the results of the present study, it seems that estrogen deficiency exerts very slight effects on the changes induced by diabetes in the rat skeletal system. This seems to agree with the lack of data on the effect of diabetes on postmenopausal osteoporosis in women. Consistently, it has been reported that, in patients, the presence of diabetes did not affect response to different antiosteoporotic drugs, regarding BMD increase and vertebral fracture risk reduction [47].

To evaluate the background of the skeletal changes observed in the present study, serum concentrations of a wide panel of cytokines was evaluated. Cytokines play major roles in numerous autoimmune diseases, hypersensitivity reactions, and pathologic conditions, like rheumatoid arthritis, dermatitis, asthma, and insulin-dependent diabetes [48]. Although cytokines mostly act locally, changes in their blood concentrations may exert general effects. It is likely that they are involved in the development of osteoporosis. Although no significant differences between the plasma levels of ten investigated cytokines in postmenopausal women with normal and low BMD were reported [49], some changes in cytokine profiles were demonstrated using advanced statistical methods [48].

The relationships between cytokines released by bone (osteoblasts, osteocytes, and osteoclasts) and immune cells are extremely complex and not fully recognized [50]. Apart from the best known RANKL/osteoprotegerin axis, the cytokines known to regulate the proliferation, differentiation, and functions of osteoblastic and osteoclastic cells, include, among others, proosteoclastogenic cytokines, stimulating bone resorption (M-CSF, TNF-*α*, IL-1, IL-6, IL-8, and IL-17), and antiosteoclastogenic cytokines (transforming growth factor *β* (TGF-*β*), IFN-*γ*, IL-4, IL-10, and IL-12) [51, 52]. A shift in general immune balance towards an activated inflammatory immune status is considered a risk factor for osteoporosis [48]. Also, in diabetes, numerous changes in cytokine levels are observed [53, 54].

In the present study, no significant effect of estrogen deficiency on the examined cytokine levels was demonstrated. It has been suggested for many years that estrogen deficiency is connected with low-grade inflammation and increased levels of proinflammatory cytokines [55, 56]. However, our results are consistent with results concerning the evaluation of ten cytokines in postmenopausal women mentioned above [49]. They are also consistent with the study of Russell et al. [57], in which a lack of effect of estradiol administration on the serum levels of most of 24 cytokines was demonstrated in estrogen-deficient rats. It is possible that the estrogen deficiency-induced effects on serum cytokine levels need more time to develop, since significant changes in the IL-1*α*, IL-6, and IFN-*γ* levels 3 months after ovariectomy were reported [58]. The diabetic rats, on the other hand, had increased levels of both proinflammatory and anti-inflammatory cytokines. The latter was probably the effect of compensatory mechanisms. It has been previously demonstrated that both estrogen deficiency and diabetes increased the bone expression of TNF-*α* mRNA in mice; the effect observed in diabetic mice was strongly intensified by estrogen deficiency [23]. Also in our study, the serum TNF-*α* concentration was the highest in the ovariectomized diabetic rats (however, the differences were insignificant).

An interesting observation of the present study is that the serum RANTES concentration was increased mainly in the ovariectomized diabetic rats. RANTES is a chemoattractant cytokine (chemokine). RANTES is, among others, secreted by osteoblasts and osteoclasts and promotes both osteoblast and osteoclast migration (with no effect on bone resorption), favoring bone formation [59]. An exceptional cytokine pattern with increased levels of RANTES and no significant effects on those of IL-6 and TNF-*α* was observed in fatty degenerative osteolysis/osteonecrosis in the jawbone [60]. Plasma RANTES has been proposed as a marker of the atherosclerotic process [61]. There is a report on the increased serum level of RANTES in osteoporotic patients [62]. It seems that the elevation of the serum RANTES concentration in the ovariectomized diabetic rats may be a compensatory reaction to strong inhibition of bone formation and an increase in bone resorption.

To better understand the interactions between diabetes- and estrogen deficiency-induced skeletal changes, the principal component analysis (PCA) was conducted on the obtained results. PCA is a multivariate technique where individual observations of variables in combined datasets are transformed into composite principal components (PCs) through data reduction. That allows for spatial differentiation of samples into clusters. The clusters separate in accordance with the differences observed in measured variables across the plane or multidimensional space built-up by two or more PCs [37, 63]. Since multiple variables take part in the PCA, their impact on the observed clustering and distance between clusters is not equal [37]. Unlike quantitative-only comparison of values for individual variables in datasets through analysis of variance (ANOVA), the PCA provides ranks which serve as an auxiliary qualitative information. The results of PCA clearly indicate significant effects of diabetes or estrogen deficiency on all the investigated groups of results. Also, differences concerning some general parameters (body mass and mass of internal organs, serum biochemical parameters) between the nonovariectomized and ovariectomized diabetic rats were observed. However, no interactions indicating differences in the skeletal or cytokine changes induced by diabetes depending on the estrogen status were demonstrated.

It must be noted that the effects of diabetes on numerous cytokine levels, regardless of the estrogen status, were very strong. Although the skeletal effects concerning the quality of bone (mechanical properties) induced by diabetes were similar to those induced by estrogen deficiency, the systemic diabetes effects, as indicated by profound changes in the cytokine levels, were much bigger.

Very strong effects of STZ-induced diabetes on cytokine levels indicate that the changes may be too profound to be relevant to those observed in diabetic patients, since patients with T1D are treated with insulin. This seems to be the main limitation of the study. However, it has been reported that, in experimental conditions, the administration of insulin improves not only glycemia but also the bone status [64]. Another limitation of the study is that it was performed on young adult rats, whereas osteoporosis is predominantly a disease of the elderly. Also, it is possible that the use of an isoflavone-free (soy-free diet), instead of a standard laboratory diet, used in the present study, might provide more significant results concerning cytokine levels in estrogen-deficient rats [57]. Nevertheless, the results of the present study indicate that the model of STZ-induced diabetes, widely used in experimental pharmacology to investigate the effects of different potential antidiabetic treatments, may be too aggressive to mirror the clinical settings.

Although the effects of type 1 and type 2 diabetes on the skeletal system in humans are differential, we observed striking similarities in the effects on bone microstructure, mechanical properties, and serum bone turnover markers in rats with experimental T1D induced by STZ and T2D induced by high-fat diet and low-dose STZ [32, 65–67]. On the other hand, similar results, concerning decreased bone turnover, to those demonstrated for ovariectomized rats with T1D [25] were obtained in ovariectomized Goto-Kakizaki rats with T2D [26]. Thus, the observations from the present study may be relevant in relation to experimental T2D.

## 5. Conclusions

In conclusion, the unfavorable skeletal changes induced by diabetes in female rats were only slightly intensified by estrogen deficiency. Despite similar effects on bone microstructure and strength, the influence of STZ-induced T1D on the skeletal system was based on much more profound changes in systemic homeostasis than those induced by estrogen deficiency.

## Figures and Tables

**Table 1:** Effects of diabetes (DM) and/or estrogen deficiency on the body mass gain and the mass of selected internal organs in rats.

Parameter/group	Nonovariectomized (NOVX) rats		Ovariectomized (OVX) rats		Two-way ANOVA		
	Control	DM	Control	DM	OVX	DM	OVXx DM
Initial body mass (g)^&^	226.9 ± 3.3	208.7 ± 3.7^∗∗^	220.9 ± 4.5	207.4 ± 3.7^∗∗∗^^X^	NS	*p* < 0.001	NS
4-week body mass gain (g)	12.1 ± 1.7	−17.4 ± 4.1^∗∗∗^	42.1 ± 5.2^∗∗∗^	−22.8 ± 5.6^∗∗∗^^XXX^	*p* < 0.01	*p* < 0.001	*p* < 0.001
Uterus mass (g)	0.511 ± 0.054	0.171 ± 0.030^∗∗∗^	0.096 ± 0.006^∗∗∗^	0.095 ± 0.007^∗∗∗^	*p* < 0.001	*p* < 0.001	*p* < 0.001
Thymus mass (g)	0.297 ± 0.019	0.093 ± 0.014^∗∗∗^	0.529 ± 0.030^∗∗∗^	0.056 ± 0.011^∗∗∗^^XXX^	*p* < 0.001	*p* < 0.001	*p* < 0.001
Liver mass (g)	5.656 ± 0.114	8.099 ± 0.329^∗∗∗^	6.197 ± 0.180	7.926 ± 0.299^∗∗∗^^XXX^	NS	*p* < 0.001	NS
Adrenals mass (g)	0.078 ± 0.004	0.071 ± 0.004	0.073 ± 0.004	0.059 ± 0.003^∗∗∗^^XX a^	*p* < 0.05	*p* < 0.01	NS

Bilateral ovariectomy was performed 5 weeks before the end of the experiment. Diabetes was induced 3 days after the ovariectomy by a single administration of streptozotocin (60 mg/kg *i.p.*). Results are presented as means ± SEM (*n* = 9 − 10). Two-way ANOVA followed by Fisher's LSD test was used for evaluation of the significance of the results. ^&^Body mass one week after the ovariectomy; ^∗∗^*p* < 0.01, ^∗∗∗^*p* < 0.001—significantly different from the NOVX control rats; ^X^*p* < 0.05, ^XX^*p* < 0.01, ^XXX^*p* < 0.001—significantly different from the OVX control rats; ^a^*p* < 0.05—significantly different from the DM-NOVX rats.

**Table 2:** Effects of diabetes (DM) and/or estrogen deficiency on the serum metabolic parameters and calcium and phosphorus concentrations.

Parameter/group	Nonovariectomized (NOVX) rats		Ovariectomized (OVX) rats		Two-way ANOVA		
	Control	DM	Control	DM	OVX	DM	OVXx DM
Glucose (mg/100 mL)	82.8 ± 2.7	561.4 ± 12.2^∗∗∗^	82.9 ± 1.5	575.9 ± 12.5^∗∗∗^^XXX^	NS	*p* < 0.001	NS
Fructosamine (mM/L)	0.483 ± 0.010	1.106 ± 0.051^∗∗∗^	0.491 ± 0.014	0.887 ± 0.111^∗∗∗^^XXX a^	NS	*p* < 0.001	NS
Triglycerides (mg/100 mL)	35.8 ± 3.8	68.8 ± 7.3^∗∗^	40.8 ± 2.2	89.1 ± 11.4^∗∗∗^^XXX^	NS	*p* < 0.001	NS
Cholesterol (mg/100 mL)	47.5 ± 4.9	53.3 ± 3.1	65.7 ± 2.4^∗∗∗^	69.8 ± 2.4^∗∗∗^^aa^	*p* < 0.001	NS	NS
LDL cholesterol (mg/100 mL)	25.0 ± 3.8	31.4 ± 4.5	49.6 ± 6.1^∗∗∗^	34.4 ± 3.9^X^	*p* < 0.01	NS	*p* < 0.05
HDL cholesterol (mg/100 mL)	28.4 ± 2.0	30.8 ± 1.3	24.2 ± 0.5	34.2 ± 3.3^XX^	NS	*p* < 0.01	NS
AOPP (*μ*mol/L)^**&**^	14.3 ± 1.4	17.2 ± 1.3	15.6 ± 1.5	23.2 ± 3.3^∗∗^^X^	NS	*p* < 0.05	NS
Calcium (mg/100 mL)	9.97 ± 0.23	11.51 ± 0.25^∗∗∗^	9.82 ± 0.27	11.86 ± 0.32^∗∗∗^^XXX^	NS	*p* < 0.001	NS
Inorganic phosphorus (mg/100 mL)	8.51 ± 0.48	4.98 ± 0.58^∗∗∗^	9.40 ± 0.51	6.38 ± 0.62^∗∗^^XXX^	*p* < 0.05	*p* < 0.001	NS

Bilateral ovariectomy was performed 5 weeks before the end of the experiment. Diabetes was induced 3 days after the ovariectomy by a single administration of streptozotocin (60 mg/kg *i.p.*). Results are presented as means ± SEM (*n* = 9 − 10). ^&^The concentration of AOPP is presented in chloramine T equivalents. Two-way ANOVA followed by Fisher's LSD test was used for evaluation of the significance of the results. ^∗∗^*p* < 0.01, ^∗∗∗^*p* < 0.001—significantly different from the NOVX control rats; ^X^*p* < 0.05, ^XX^*p* < 0.01, ^XXX^*p* < 0.001—significantly different from the OVX control rats; ^a^*p* < 0.05, ^aa^*p* < 0.01—significantly different from the DM-NOVX rats.

**Table 3:** Effects of diabetes (DM) and/or estrogen deficiency on the bone mass, macrometric parameters, composition, and mineralization in the femur and L4 vertebra.

Parameter/group		Nonovariectomized (NOVX) rats		Ovariectomized (OVX) rats		Two-way ANOVA		
		Control	DM	Control	DM	OVX	DM	OVXx DM
Bone mass (g)	Femur	0.682 ± 0.014	0.629 ± 0.012^∗∗^	0.677 ± 0.014	0.618 ± 0.012^∗∗^^XX^	NS	*p* < 0.001	NS
	L4 vertebra	0.214 ± 0.009	0.188 ± 0.008^∗^	0.214 ± 0.006	0.178 ± 0.005^∗∗∗^^XX^	NS	*p* < 0.001	NS
Femoral length (mm)		33.54 ± 0.18	32.83 ± 0.16^∗^	34.07 ± 0.22	32.80 ± 0.20^∗∗^^XXX^	NS	*p* < 0.001	NS
Femoral diameter (mm)		3.36 ± 0.03	3.28 ± 0.03	3.37 ± 0.04	3.26 ± 0.04	NS	*p* < 0.01	NS
Bone mineral mass (g)	Femur	0.331 ± 0.007	0.293 ± 0.007^∗∗∗^	0.320 ± 0.006	0.283 ± 0.005^∗∗∗^^XXX^	NS	*p* < 0.001	NS
	L4 vertebra	0.086 ± 0.003	0.073 ± 0.003^∗∗∗^	0.086 ± 0.002	0.068 ± 0.002^∗∗∗^^XXX^	NS	*p* < 0.001	NS
Bone mineral content (g/g)	Femur	0.485 ± 0.003	0.466 ± 0.006^∗∗^	0.473 ± 0.004^∗^	0.458 ± 0.003^∗∗∗^^XX^	*p* < 0.05	*p* < 0.001	NS
	L4 vertebra	0.407 ± 0.011	0.391 ± 0.008	0.403 ± 0.007	0.384 ± 0.005	NS	*p* < 0.05	NS
Bone water content (g/g)	Femur	0.276 ± 0.004	0.294 ± 0.007	0.303 ± 0.013	0.301 ± 0.004	*p* < 0.05	NS	NS
	L4 vertebra	0.345 ± 0.014	0.362 ± 0.010	0.347 ± 0.007	0.362 ± 0.009	NS	NS	NS
Bone organic substances content (g/g)	Femur	0.239 ± 0.002	0.240 ± 0.002	0.224 ± 0.012	0.241 ± 0.002	NS	NS	NS
	L4 vertebra	0.248 ± 0.004	0.248 ± 0.003	0.250 ± 0.002	0.254 ± 0.006	NS	NS	NS
Calcium content (g/g of bone mineral)	Femur	0.373 ± 0.007	0.364 ± 0.012	0.363 ± 0.007	0.377 ± 0.006	NS	NS	NS
	L4 vertebra	0.345 ± 0.018	0.347 ± 0.016	0.346 ± 0.015	0.339 ± 0.015	NS	NS	NS
Phosphorus content (g/g of bone mineral)	Femur	0.176 ± 0.003	0.173 ± 0.002	0.171 ± 0.002	0.180 ± 0.002^XX a^	NS	NS	*p* < 0.01
	L4 vertebra	0.156 ± 0.004	0.155 ± 0.003	0.158 ± 0.004	0.153 ± 0.003	NS	NS	NS

Bilateral ovariectomy was performed 5 weeks before the end of the experiment. Diabetes was induced 3 days after the ovariectomy by a single administration of streptozotocin (60 mg/kg *i.p.*). Results are presented as means ± SEM (*n* = 9 − 10). Two-way ANOVA followed by Fisher's LSD test was used for evaluation of the significance of the results. ^∗^*p* < 0.05, ^∗∗^*p* < 0.01, ^∗∗∗^*p* < 0.001—significantly different from the NOVX control rats; ^XX^*p* < 0.01, ^XXX^*p* < 0.001—significantly different from the OVX control rats; ^a^*p* < 0.05—significantly different from the DM-NOVX rats.

**Table 4:** Effects of diabetes (DM) and/or estrogen deficiency on the histomorphometric parameters of compact bone of the femoral diaphysis in rats.

Parameter/group		Nonovariectomized (NOVX) rats		Ovariectomized (OVX) rats		Two-way ANOVA		
		Control	DM	Control	DM	OVX	DM	OVXx DM
Diaphysis	Tt.Ar (mm^2^)	8.21 ± 0.11	7.81 ± 0.16	8.39 ± 0.18	7.79 ± 0.13^XX^	NS	*p* < 0.01	NS
	Ct.Ar (mm^2^)	5.42 ± 0.06	5.10 ± 0.08^∗∗^	5.51 ± 0.08	5.02 ± 0.05^∗∗∗^^XXX^	NS	*p* < 0.001	NS
	Ma.Ar (mm^2^)	2.79 ± 0.07	2.70 ± 0.11	2.87 ± 0.13	2.78 ± 0.10	NS	NS	NS
	Ma.Ar/Tt.Ar	0.340 ± 0.005	0.346 ± 0.008	0.341 ± 0.009	0.355 ± 0.007	NS	NS	NS

Bilateral ovariectomy was performed 5 weeks before the end of the experiment. Diabetes was induced 3 days after the ovariectomy by a single administration of streptozotocin (60 mg/kg *i.p.*). Results are presented as means ± SEM (*n* = 9 − 10). Two-way ANOVA followed by Fisher's LSD test was used for evaluation of the significance of the results. ^∗∗^*p* < 0.01, ^∗∗∗^*p* < 0.001—significantly different from the NOVX control rats; ^XX^*p* < 0.01, ^XXX^*p* < 0.001—significantly different from the OVX control rats; Tt.Ar: transverse cross-section area of the whole diaphysis; Ct.Ar: transverse cross-section area of the cortical bone; Ma.Ar: transverse cross-section area of the marrow cavity; Ma.Ar/Tt.Ar: transverse cross-section area of the marrow cavity/diaphysis ratio.

**Table 5:** Effects of diabetes (DM) and/or estrogen deficiency on the mechanical properties of cancellous (the proximal tibial metaphysis) and compact (the femoral diaphysis) bone in rats.

Parameter/group		Nonovariectomized (NOVX) rats		Ovariectomized (OVX) rats		Two-way ANOVA		
		Control	DM	Control	DM	OVX	DM	OVXx DM
Tibia	Young's modulus (MPa)	4063 ± 430	3269 ± 390	2713 ± 248^∗∗^	3169 ± 182	*p* < 0.05	NS	NS
	Fracture point load (N)	89.5 ± 7.5	54.5 ± 5.0^∗∗∗^	60.0 ± 2.8^∗∗∗^	43.6 ± 2.2^∗∗∗^^X^	*p* < 0.001	*p* < 0.001	NS
	Displacement for fracture point load (mm)	1.068 ± 0.054	1.004 ± 0.088	1.208 ± 0.074	0.953 ± 0.087	NS	*p* < 0.05	NS
	Energy for fracture point load (mJ)	80.8 ± 5.7	49.9 ± 7.0^∗∗∗^	63.9 ± 5.0^∗^	40.7 ± 3.9^∗∗∗^^XX^	*p* < 0.05	*p* < 0.001	NS
	Stress for fracture point load (MPa)	74.1 ± 7.8	47.3 ± 4.8^∗∗∗^	47.4 ± 4.2^∗∗∗^	35.6 ± 1.3^∗∗∗^	*p* < 0.001	*p* < 0.001	NS

Femur	Young's modulus (MPa)	8929 ± 242	10886 ± 373^∗∗∗^	8957 ± 418	9955 ± 333^∗^^X^	NS	*p* < 0.001	NS
	Maximum load (N)	124.8 ± 4.3	125.8 ± 3.8	130.1 ± 3.7	119.3 ± 5.3	NS	NS	NS
	Displacement for maximum load (mm)	0.502 ± 0.021	0.493 ± 0.019	0.516 ± 0.034	0.487 ± 0.016	NS	NS	NS
	Energy for maximum load (mJ)	38.0 ± 2.8	38.2 ± 2.4	41.0 ± 3.7	35.7 ± 2.6	NS	NS	NS
	Stress for maximum load (MPa)	168.9 ± 4.2	189.7 ± 4.9^∗∗^	171.2 ± 4.3	174.6 ± 5.7^a^	NS	*p* < 0.05	NS
	Fracture point load (N)	123.3 ± 4.4	124.8 ± 3.6	129.4 ± 3.8	118.3 ± 5.2	NS	NS	NS
	Displacement for fracture point load (mm)	0.517 ± 0.021	0.505 ± 0.021	0.524 ± 0.034	0.501 ± 0.019	NS	NS	NS
	Energy for fracture point load (mJ)	39.8 ± 2.9	39.8 ± 2.6	42.0 ± 3.8	37.5 ± 3.2	NS	NS	NS
	Stress for fracture point load (MPa)	166.8 ± 4.6	188.1 ± 4.6^∗∗^	170.3 ± 4.4	173.1 ± 5.7^a^	NS	*p* < 0.05	NS

Bilateral ovariectomy was performed 5 weeks before the end of the experiment. Diabetes was induced 3 days after the ovariectomy by a single administration of streptozotocin (60 mg/kg *i.p.*). Results are presented as means ± SEM (*n* = 9 − 10). Two-way ANOVA followed by Fisher's LSD test was used for evaluation of the significance of the results. ^∗^*p* < 0.05, ^∗∗^*p* < 0.01, ^∗∗∗^*p* < 0.001—significantly different from the NOVX control rats; ^X^*p* < 0.05, ^XX^*p* < 0.01—significantly different from the OVX control rats; ^a^*p* < 0.05—significantly different from the DM-NOVX rats.

**Table 6:** Effects of diabetes (DM) and/or estrogen deficiency on the serum concentrations of cytokines in rats.

Parameter/group	Nonovariectomized (NOVX) rats		Ovariectomized (OVX) rats		Two-way ANOVA		
	Control	DM	Control	DM	OVX	DM	OVXx DM
IL-1*α* (pg/mL)	174.0 ± 25.7	366.7 ± 64.9^∗∗^	180.3 ± 34.9	362.1 ± 42.9^∗∗^^XX^	NS	*p* < 0.001	NS
IL-1*β* (pg/mL)	613.6 ± 155.9	43675 ± 23833	495.5 ± 138.5	29915 ± 22082	NS	*p* < 0.05	NS
IL-2 (pg/mL)	585.7 ± 260.4	2670 ± 1394	419.3 ± 141.9	743.9 ± 377.8	NS	NS	NS
IL-4 (pg/mL)	105.9 ± 19.9	233.7 ± 37.8^∗∗^	106.6 ± 22.9	152.3 ± 19.9^a^	NS	*p* < 0.01	NS
IL-5 (pg/mL)	565.7 ± 39.6	649.2 ± 42.1	543.4 ± 49.2	610.9 ± 78.6	NS	NS	NS
IL-6 (pg/mL)	470.5 ± 214.2	750.6 ± 185.5	379.1 ± 86.4	635.2 ± 158.5	NS	NS	NS
IL-7 (pg/mL)	506.5 ± 108.7	5886 ± 1750^∗∗^	426.7 ± 91.8	5168 ± 1873^∗^^X^	NS	*p* < 0.001	NS
IL-10 (pg/mL)	84.8 ± 15.0	193.2 ± 33.5^∗∗^	80.8 ± 18.0	170.8 ± 29.3^∗^^X^	NS	*p* < 0.001	NS
IL-12p70 (pg/mL)	250.4 ± 62.7	307.5 ± 57.7	274.3 ± 92.3	324.2 ± 75.2	NS	NS	NS
IL-13 (pg/mL)	139.2 ± 25.5	424.1 ± 140.4	128.2 ± 35.1	209.2 ± 63.7	NS	*p* < 0.05	NS
IL-17A (pg/mL)	62.3 ± 9.0	95.6 ± 19.7	61.9 ± 12.7	110.4 ± 19.2	NS	*p* < 0.05	NS
IL-18 (pg/mL)	3950 ± 851	10802 ± 5377	4341 ± 208	4663 ± 1651	NS	NS	NS
G-CSF (pg/ml)	2.16 ± 0.77	5.98 ± 2.07	1.56 ± 0.72	5.45 ± 1.65	NS	*p* < 0.05	NS
GM-CSF (pg/ml)	874.2 ± 224.5	26206 ± 7878^∗∗^	793.5 ± 216.4	22875 ± 6940^∗∗^^XX^	NS	*p* < 0.001	NS
GRO/KC (pg/ml)	627.1 ± 65.4	12186 ± 6622	659.9 ± 178.6	6295 ± 2398	NS	*p* < 0.05	NS
IFN-*γ* (pg/ml)	308.6 ± 50.8	618.8 ± 146.8	293.8 ± 76.3	459.3 ± 87.4	NS	*p* < 0.05	NS
M-CSF (pg/ml)	22.8 ± 5.4	55.3 ± 12.2^∗∗^	15.8 ± 3.8	51.7 ± 8.4^∗^^XX^	NS	*p* < 0.001	NS
MIP-1a (pg/ml)	206.8 ± 56.5	10541 ± 2459^∗∗∗^	157.7 ± 35.3	10720 ± 2032^∗∗∗^^XXX^	NS	*p* < 0.001	NS
MIP-3a (pg/ml)	28.6 ± 4.1	106.5 ± 13.9	26.3 ± 4.9	206.4 ± 67.2^∗∗^^XX^	NS	*p* < 0.01	NS
RANTES (pg/ml)	2011 ± 214	2192 ± 223	2204 ± 813	8095 ± 1414^∗∗∗^^XXX aaa^	*p* < 0.01	*p* < 0.01	*p* < 0.01
TNF-*α* (pg/ml)	326.4 ± 108.6	457.4 ± 88.1	254.4 ± 81.3	623.0 ± 127.6	NS	*p* < 0.05	NS
VEGF (pg/ml)	486.8 ± 67.0	7360 ± 2916^∗∗^	608.9 ± 122.2	4020 ± 991	NS	*p* < 0.01	NS
MCP-1 (MCAF) (pg/ml)	3119 ± 493	834106 ± 241089^∗∗∗^	4110 ± 1334	598440 ± 159609^∗∗^^XX^	NS	*p* < 0.001	NS

Bilateral ovariectomy was performed 5 weeks before the end of the experiment. Diabetes was induced 3 days after the ovariectomy by a single administration of streptozotocin (60 mg/kg *i.p.*). Results are presented as means ± SEM (*n* = 7). Two-way ANOVA followed by Fisher's LSD test was used for evaluation of the significance of the results. ^∗^*p* < 0.05, ^∗∗^*p* < 0.01, ^∗∗∗^*p* < 0.001—significantly different from the NOVX control rats; ^X^*p* < 0.05, ^XX^*p* < 0.01, ^XXX^*p* < 0.001—significantly different from the OVX control rats; ^a^*p* < 0.05, ^aaa^*p* < 0.001—significantly different from the DM-NOVX rats.

## Data Availability

The data used to support the findings of this study are available from the corresponding author upon request.

## Abbreviations

ANOVA: Analysis of variance
AOPP: Advanced oxidation protein products
ASBMR: American Society for Bone and Mineral Research
BMD: Bone mineral density
BV/TV: Bone volume to tissue volume ratio
Ct.Ar: Transverse cross-sectional area of the cortical bone
CTX-I: C-terminal telopeptide fragments of type I collagen
DM: Diabetes mellitus
G-CSF: Granulocyte colony-stimulating factor
GM-CSF: Granulocyte-macrophage colony-stimulating factor
GRO/KC: Growth-regulated oncogene/keratinocyte chemoattractant
HDL: High-density lipoprotein
IFN-*γ*: Interferon-*γ*
IL: Interleukin
LDL: Low-density lipoprotein
LSD: Least significant difference
Ma.Ar: Transverse cross-sectional area of the marrow cavity
Ma.Ar/Tt.Ar: Transverse cross-sectional area of the marrow cavity/total diaphysis area ratio
MANOVA: Multivariate analysis of variance
MAR: Mineral apposition rate
M-CSF: Macrophage colony-stimulating factor
MCP-1 (MCAF): Monocyte chemoattractant protein-1 (monocyte chemotactic and activating factor)
MIP-1a: Macrophage inflammatory protein-1a
MIP-3a: Macrophage inflammatory protein-3a
NOVX: Nonovariectomized
NS: Not significant
OVX: Ovariectomized
PCA: Principal component analysis
RANKL: Receptor activator of nuclear factor *κ*B ligand
RANTES: Regulated on activation, normal T cell expressed and secreted
SEM: Standard error of the mean
STZ: Streptozotocin
T1D: Type 1 diabetes
T2D: Type 2 diabetes
Tb.N: Trabecular number
Tb.Sp: Trabecular separation
Tb.Th: Trabecular thickness
TGF-*β*: Transforming growth factor *β*
TNF-*α*: Tumor necrosis factor *α*
Tt.Ar: Transverse cross-sectional area of the whole diaphysis
VEGF: Vascular endothelial growth factor.

## Supplementary Materials

Supplementary Materials Supplementary Material 1 presents results for two-way MANOVA for the PCA biplots obtained for individual datasets concerning results presented in Figures 1 and 2, Figures 4–6, Figures 8 and 9, and Figures 11–13.
