# Supplementary material for: Negligible Effect of Estrogen Deficiency on Development of Skeletal Changes Induced by Type 1 Diabetes in Experimental Rat Models
Source: Mediators Inflamm. 2020 Nov 6;2020:2793804. doi: 10.1155/2020/2793804 (PMC7665927; doi:10.1155/2020/2793804)
Supplement: Supplementary Materials — Supplementary Material 1 presents results for two-way MANOVA for the PCA biplots obtained for individual datasets concerning results presented in Figures 1 and 2, Figures 4–6, Figures 8 and 9, and Figures 11–13. [file 2793804.f1.docx]

**Supplementary material 1**

**1A**—Two-way MANOVA from PCA biplot of the results concerning the body mass gain and the mass of internal organs (uterus, thymus, adrenal glands, liver) in the diabetic and/or estrogen-deficient rats.

MANOVA (PCA coordinates):

| Effect | Test | Value | F | Effect df | Error df | *p* |
| --- | --- | --- | --- | --- | --- | --- |
| OVX | Wilks | 0.289 | 41.915 | 2 | 34 | <0.001 |
| DM | Wilks | 0.061 | 259.453 | 2 | 34 | <0.001 |
| OVXxDM | Wilks | 0.339 | 33.151 | 2 | 34 | <0.001 |

Axes individually:

| Effect | df | PC1 | | | | PC2 | | | |
| --- | --- | --- | --- | --- | --- | --- | --- | --- | --- |
|  |  | SS | MS | F | *p* | SS | MS | F | *p* |
| OVX | 1 | 0.104 | 0.104 | 0.580 | 0.451 | 22.461 | 22.461 | 83.161 | <0.001 |
| DM | 1 | 89.375 | 89.375 | 497.218 | <0.001 | 0.009 | 0.009 | 0.032 | 0.859 |
| OVXxDM | 1 | 2.022 | 2.022 | 11.252 | <0.01 | 10.929 | 10.929 | 40.466 | <0.001 |
| Error | 35 | 6.291 | 0.180 |  |  | 9.453 | 0.270 |  |  |

LSD *post-hoc* (*p* values):

| **PC1** | | NOVX | DM-NOVX | OVX | DM-OVX |
| --- | --- | --- | --- | --- | --- |
| NOVX | |  | <0.001 | 0.072 | <0.001 |
| DM-NOVX | | <0.001 |  | <0.001 | <0.01 |
| OVX | | 0.072 | <0.001 |  | <0.001 |
| DM-OVX | | <0.001 | <0.01 | <0.001 |  |
|  |  |  |  |  |  |
| **PC2** | | NOVX | DM-NOVX | OVX | DM-OVX |
| NOVX | |  | <0.001 | <0.001 | <0.001 |
| DM-NOVX | | <0.001 |  | <0.001 | 0.062 |
| OVX | | <0.001 | <0.001 |  | <0.001 |
| DM-OVX | | <0.001 | 0.062 | <0.001 |  |

Significant results marked in red;
df—degrees of freedom, SS—sum of squares, MS—mean squares, F—F statistic, *p*—*p* value

**1B**—Two-way MANOVA from PCA biplot of the results concerning the concentrations of the serum biochemical metabolic parameters in the diabetic and/or estrogen-deficient rats.

MANOVA (PCA coordinates):

| Effect | Test | Value | F | Effect df | Error df | *p* |
| --- | --- | --- | --- | --- | --- | --- |
| OVX | Wilks | 0.632 | 9.895 | 2 | 34 | <0.001 |
| DM | Wilks | 0.086 | 180.638 | 2 | 34 | <0.001 |
| OVXxDM | Wilks | 0.993 | 0.123 | 2 | 34 | 0.885 |

Axes individually:

| Effect | df | PC1 | | | | PC2 | | | |
| --- | --- | --- | --- | --- | --- | --- | --- | --- | --- |
|  |  | SS | MS | F | *p* | SS | MS | F | *p* |
| OVX | 1 | 3.607 | 3.607 | 5.814 | <0.05 | 20.400 | 20.400 | 20.293 | <0.001 |
| DM | 1 | 124.412 | 124.412 | 200.528 | <0.001 | 5.369 | 5.369 | 5.341 | <0.05 |
| OVXxDM | 1 | 0.056 | 0.056 | 0.090 | 0.766 | 0.023 | 0.023 | 0.023 | 0.881 |
| Error | 35 | 21.715 | 0.620 |  |  | 35.185 | 1.005 |  |  |

LSD *post-hoc* (*p* values):

| **PC1** | | NOVX | DM-NOVX | OVX | DM-OVX |
| --- | --- | --- | --- | --- | --- |
| NOVX | |  | <0.001 | 0.139 | <0.001 |
| DM-NOVX | | <0.001 |  | <0.001 | 0.067 |
| OVX | | 0.139 | <0.001 |  | <0.001 |
| DM-OVX | | <0.001 | 0.067 | <0.001 |  |
|  |  |  |  |  |  |
| **PC2** | | NOVX | DM-NOVX | OVX | DM-OVX |
| NOVX | |  | 0.141 | <0.01 | 0.125 |
| DM-NOVX | | 0.141 |  | <0.001 | <0.01 |
| OVX | | <0.01 | <0.001 |  | 0.086 |
| DM-OVX | | 0.125 | <0.01 | 0.086 |  |

Significant results marked in red;
df—degrees of freedom, SS—sum of squares, MS—mean squares, F—F statistic, *p*—*p* value

**1C**—Two-way MANOVA from PCA biplot of the results concerning the concentrations of the serum bone turnover markers and concentrations of calcium and inorganic phosphorus in the diabetic and/or estrogen-deficient rats.

MANOVA (PCA coordinates):

| Effect | Test | Value | F | Effect df | Error df | *p* |
| --- | --- | --- | --- | --- | --- | --- |
| OVX | Wilks | 0.779 | 4.811 | 2 | 34 | <0.05 |
| DM | Wilks | 0.229 | 57.218 | 2 | 34 | <0.001 |
| OVXxDM | Wilks | 0.958 | 0.747 | 2 | 34 | 0.482 |

Axes individually:

| Effect | df | PC1 | | | | PC2 | | | |
| --- | --- | --- | --- | --- | --- | --- | --- | --- | --- |
|  |  | SS | MS | F | *p* | SS | MS | F | *p* |
| OVX | 1 | 1.969 | 1.969 | 2.729 | 0.107 | 3.897 | 3.897 | 8.158 | <0.01 |
| DM | 1 | 81.705 | 81.705 | 113.257 | <0.001 | 0.348 | 0.348 | 0.727 | 0.400 |
| OVXxDM | 1 | 0.936 | 0.936 | 1.297 | 0.262 | 0.185 | 0.185 | 0.386 | 0.538 |
| Error | 35 | 25.249 | 0.721 |  |  | 16.722 | 0.478 |  |  |

LSD *post-hoc* (*p* values):

| **PC1** | | NOVX | DM-NOVX | OVX | DM-OVX |
| --- | --- | --- | --- | --- | --- |
| NOVX | |  | <0.001 | 0.053 | <0.001 |
| DM-NOVX | | <0.001 |  | <0.001 | 0.722 |
| OVX | | 0.053 | <0.001 |  | <0.001 |
| DM-OVX | | <0.001 | 0.722 | <0.001 |  |
|  |  |  |  |  |  |
| **PC2** | | NOVX | DM-NOVX | OVX | DM-OVX |
| NOVX | |  | 0.311 | <0.05 | <0.05 |
| DM-NOVX | | 0.311 |  | 0.171 | 0.128 |
| OVX | | <0.05 | 0.171 |  | 0.869 |
| DM-OVX | | <0.05 | 0.128 | 0.869 |  |

Significant results marked in red;
df—degrees of freedom, SS—sum of squares, MS—mean squares, F—F statistic, *p*—*p* value

**1D**—Two-way MANOVA from PCA biplot of the results concerning the bone mass and macrometric parameters in the diabetic and/or estrogen-deficient rats.

MANOVA (PCA coordinates):

| Effect | Test | Value | F | Effect df | Error df | *p* |
| --- | --- | --- | --- | --- | --- | --- |
| OVX | Wilks | 0.991 | 0.148 | 2 | 34 | 0.863 |
| DM | Wilks | 0.621 | 10.393 | 2 | 34 | <0.001 |
| OVXxDM | Wilks | 0.973 | 0.472 | 2 | 34 | 0.628 |

Axes individually:

| Effect | df | PC1 | | | | PC2 | | | |
| --- | --- | --- | --- | --- | --- | --- | --- | --- | --- |
|  |  | SS | MS | F | *p* | SS | MS | F | *p* |
| OVX | 1 | 0.228 | 0.228 | 0.069 | 0.794 | 0.175 | 0.175 | 0.223 | 0.640 |
| DM | 1 | 69.229 | 69.229 | 21.123 | <0.001 | 0.068 | 0.068 | 0.087 | 0.770 |
| OVXxDM | 1 | 2.987 | 2.987 | 0.911 | 0.346 | 0.031 | 0.031 | 0.039 | 0.844 |
| Error | 35 | 114.709 | 3.277 |  |  | 27.569 | 0.788 |  |  |

LSD *post-hoc* (*p* values):

| **PC1** | | NOVX | DM-NOVX | OVX | DM-OVX |
| --- | --- | --- | --- | --- | --- |
| NOVX | |  | <0.05 | 0.623 | <0.01 |
| DM-NOVX | | <0.05 |  | <0.01 | 0.401 |
| OVX | | 0.623 | <0.01 |  | <0.001 |
| DM-OVX | | <0.01 | 0.401 | <0.001 |  |
|  |  |  |  |  |  |

Significant results marked in red;
df—degrees of freedom, SS—sum of squares, MS—mean squares, F—F statistic, *p*—*p* value

**1E**—Two-way MANOVA from PCA biplot of the results concerning the bone composition and mineralization in the diabetic and/or estrogen-deficient rats.

MANOVA (PCA coordinates):

| Effect | Test | Value | F | Effect df | Error df | *p* |
| --- | --- | --- | --- | --- | --- | --- |
| OVX | Wilks | 0.901 | 1.861 | 2 | 34 | 0.171 |
| DM | Wilks | 0.771 | 5.059 | 2 | 34 | <0.05 |
| OVXxDM | Wilks | 0.927 | 1.341 | 2 | 34 | 0.275 |

Axes individually:

| Effect | df | PC1 | | | | PC2 | | | |
| --- | --- | --- | --- | --- | --- | --- | --- | --- | --- |
|  |  | SS | MS | F | *p* | SS | MS | F | *p* |
| OVX | 1 | 14.137 | 14.137 | 3.791 | 0.060 | 0.014 | 0.014 | 0.005 | 0.941 |
| DM | 1 | 5.217 | 5.217 | 1.399 | 0.245 | 22.126 | 22.126 | 8.534 | <0.01 |
| OVXxDM | 1 | 6.953 | 6.953 | 1.865 | 0.181 | 2.759 | 2.759 | 1.064 | 0.309 |
| Error | 35 | 130.506 | 3.729 |  |  | 90.741 | 2.593 |  |  |

LSD *post-hoc* (*p* values):

| **PC2** | | NOVX | DM-NOVX | OVX | DM-OVX |
| --- | --- | --- | --- | --- | --- |
| NOVX | |  | 0.196 | 0.497 | <0.05 |
| DM-NOVX | | 0.196 |  | 0.055 | 0.446 |
| OVX | | 0.497 | 0.055 |  | <0.01 |
| DM-OVX | | <0.05 | 0.446 | <0.01 |  |
|  |  |  |  |  |  |

Significant results marked in red;
df—degrees of freedom, SS—sum of squares, MS—mean squares, F—F statistic, *p*—*p* value

**1F**—Two-way MANOVA from PCA biplot of the results concerning histomorphometric parameters of cancellous bone (the distal femoral metaphysis) in the diabetic and/or estrogen-deficient rats.

MANOVA (PCA coordinates):

| Effect | Test | Value | F | Effect df | Error df | *p* |
| --- | --- | --- | --- | --- | --- | --- |
| OVX | Wilks | 0.876 | 2.412 | 2 | 34 | 0.105 |
| DM | Wilks | 0.665 | 8.566 | 2 | 34 | <0.001 |
| OVXxDM | Wilks | 0.866 | 2.637 | 2 | 34 | 0.086 |

Axes individually:

| Effect | df | PC1 | | | | PC2 | | | |
| --- | --- | --- | --- | --- | --- | --- | --- | --- | --- |
|  |  | SS | MS | F | *p* | SS | MS | F | *p* |
| OVX | 1 | 10.080 | 10.080 | 3.725 | 0.062 | 2.657 | 2.657 | 1.351 | 0.253 |
| DM | 1 | 6.201 | 6.201 | 2.292 | 0.139 | 29.558 | 29.558 | 15.033 | <0.001 |
| OVXxDM | 1 | 10.070 | 10.070 | 3.722 | 0.062 | 3.611 | 3.611 | 1.837 | 0.184 |
| Error | 35 | 94.699 | 2.706 |  |  | 68.820 | 1.966 |  |  |

LSD *post-hoc* (*p* values):

| **PC2** | | NOVX | DM-NOVX | OVX | DM-OVX |
| --- | --- | --- | --- | --- | --- |
| NOVX | |  | 0.087 | 0.080 | 0.060 |
| DM-NOVX | | 0.087 |  | <0.01 | 0.894 |
| OVX | | 0.080 | <0.01 |  | <0.001 |
| DM-OVX | | 0.060 | 0.894 | <0.001 |  |
|  |  |  |  |  |  |

Significant results marked in red;
df—degrees of freedom, SS—sum of squares, MS—mean squares, F—F statistic, *p*—*p* value

**1G**—Two-way MANOVA from PCA biplot of the results concerning histomorphometric parameters of compact bone (the tibial and femoral diaphysis) in the diabetic and/or estrogen-deficient rats.

MANOVA (PCA coordinates):

| Effect | Test | Value | F | Effect df | Error df | *p* |
| --- | --- | --- | --- | --- | --- | --- |
| OVX | Wilks | 0.996 | 0.064 | 2 | 34 | 0.938 |
| DM | Wilks | 0.784 | 4.680 | 2 | 34 | <0.05 |
| OVXxDM | Wilks | 0.973 | 0.474 | 2 | 34 | 0.626 |

Axes individually:

| Effect | df | PC1 | | | | PC2 | | | |
| --- | --- | --- | --- | --- | --- | --- | --- | --- | --- |
|  |  | SS | MS | F | *p* | SS | MS | F | *p* |
| OVX | 1 | 0.458 | 0.458 | 0.110 | 0.742 | 0.072 | 0.072 | 0.035 | 0.853 |
| DM | 1 | 25.527 | 25.527 | 6.121 | <0.05 | 5.083 | 5.083 | 2.450 | 0.127 |
| OVXxDM | 1 | 0.478 | 0.478 | 0.115 | 0.737 | 1.612 | 1.612 | 0.777 | 0.384 |
| Error | 35 | 145.976 | 4.171 |  |  | 72.618 | 2.075 |  |  |

LSD *post-hoc* (*p* values):

| **PC1** | | NOVX | DM-NOVX | OVX | DM-OVX |
| --- | --- | --- | --- | --- | --- |
| NOVX | |  | 0.145 | 0.634 | 0.134 |
| DM-NOVX | | 0.145 |  | 0.058 | 0.996 |
| OVX | | 0.634 | 0.058 |  | 0.052 |
| DM-OVX | | 0.134 | 0.996 | 0.052 |  |
|  |  |  |  |  |  |

Significant results marked in red;
df—degrees of freedom, SS—sum of squares, MS—mean squares, F—F statistic, *p*—*p* value

**1H**—Two-way MANOVA from PCA biplot of the results concerning mechanical properties of cancellous bone (the proximal tibial metaphysis) in the diabetic and/or estrogen-deficient rats.

MANOVA (PCA coordinates):

| Effect | Test | Value | F | Effect df | Error df | *p* |
| --- | --- | --- | --- | --- | --- | --- |
| OVX | Wilks | 0.642 | 9.493 | 2 | 34 | <0.001 |
| DM | Wilks | 0.476 | 18.706 | 2 | 34 | <0.001 |
| OVXxDM | Wilks | 0.859 | 2.781 | 2 | 34 | 0.076 |

Axes individually:

| Effect | df | PC1 | | | | PC2 | | | |
| --- | --- | --- | --- | --- | --- | --- | --- | --- | --- |
|  |  | SS | MS | F | *p* | SS | MS | F | *p* |
| OVX | 1 | 59.257 | 59.257 | 19.545 | <0.001 | 1.022 | 1.022 | 0.390 | 0.537 |
| DM | 1 | 93.033 | 93.033 | 30.686 | <0.001 | 10.255 | 10.255 | 3.909 | 0.056 |
| OVXxDM | 1 | 9.496 | 9.496 | 3.132 | 0.085 | 8.947 | 8.947 | 3.411 | 0.073 |
| Error | 35 | 106.113 | 3.032 |  |  | 91.821 | 2.623 |  |  |

LSD *post-hoc* (*p* values):

| **PC1** | | NOVX | DM-NOVX | OVX | DM-OVX |
| --- | --- | --- | --- | --- | --- |
| NOVX | |  | <0.001 | <0.001 | <0.001 |
| DM-NOVX | | <0.001 |  | 0.440 | 0.073 |
| OVX | | <0.001 | 0.440 |  | <0.05 |
| DM-OVX | | <0.001 | 0.073 | <0.05 |  |
|  |  |  |  |  |  |

Significant results marked in red;
df—degrees of freedom, SS—sum of squares, MS—mean squares, F—F statistic, *p*—*p* value

**1I**—Two-way MANOVA from PCA biplot of the results concerning mechanical properties of compact bone (the femoral diaphysis) in the diabetic and/or estrogen-deficient rats.

MANOVA (PCA coordinates):

| Effect | Test | Value | F | Effect df | Error df | *p* |
| --- | --- | --- | --- | --- | --- | --- |
| OVX | Wilks | 0.949 | 0.916 | 2 | 34 | 0.410 |
| DM | Wilks | 0.742 | 5.902 | 2 | 34 | <0.01 |
| OVXxDM | Wilks | 0.932 | 1.242 | 2 | 34 | 0.302 |

Axes individually:

| Effect | df | PC1 | | | | PC2 | | | |
| --- | --- | --- | --- | --- | --- | --- | --- | --- | --- |
|  |  | SS | MS | F | *p* | SS | MS | F | *p* |
| OVX | 1 | 0.849 | 0.849 | 0.147 | 0.703 | 4.119 | 4.119 | 1.708 | 0.200 |
| DM | 1 | 0.127 | 0.127 | 0.022 | 0.883 | 29.305 | 29.305 | 12.149 | <0.01 |
| OVXxDM | 1 | 8.262 | 8.262 | 1.435 | 0.239 | 2.528 | 2.528 | 1.048 | 0.313 |
| Error | 35 | 201.538 | 5.758 |  |  | 84.421 | 2.412 |  |  |

LSD *post-hoc* (*p* values):

| **PC2** | | NOVX | DM-NOVX | OVX | DM-OVX |
| --- | --- | --- | --- | --- | --- |
| NOVX | |  | <0.01 | 0.840 | 0.127 |
| DM-NOVX | | <0.01 |  | <0.01 | 0.113 |
| OVX | | 0.840 | <0.01 |  | 0.086 |
| DM-OVX | | 0.127 | 0.113 | 0.086 |  |
|  |  |  |  |  |  |

Significant results marked in red;
df—degrees of freedom, SS—sum of squares, MS—mean squares, F—F statistic, *p*—*p* value

**1J**—Two-way MANOVA from PCA biplot of the results concerning the serum concentrations of 23 cytokines in the diabetic and/or estrogen-deficient rats.

MANOVA (PCA coordinates):

| Effect | Test | Value | F | Effect df | Error df | *p* |
| --- | --- | --- | --- | --- | --- | --- |
| OVX | Wilks | 0.978 | 0.389 | 2 | 34 | 0.681 |
| DM | Wilks | 0.474 | 18.853 | 2 | 34 | <0.001 |
| OVXxDM | Wilks | 0.984 | 0.274 | 2 | 34 | 0.762 |

Axes individually:

| Effect | df | PC1 | | | | PC2 | | | |
| --- | --- | --- | --- | --- | --- | --- | --- | --- | --- |
|  |  | SS | MS | F | *p* | SS | MS | F | *p* |
| OVX | 1 | 2.637 | 2.637 | 0.459 | 0.502 | 0.695 | 0.695 | 0.109 | 0.743 |
| DM | 1 | 123.554 | 123.554 | 21.527 | <0.001 | 37.008 | 37.008 | 5.817 | <0.05 |
| OVXxDM | 1 | 1.287 | 1.287 | 0.224 | 0.639 | 1.000 | 1.000 | 0.157 | 0.694 |
| Error | 35 | 200.879 | 5.739 |  |  | 222.654 | 6.362 |  |  |

LSD *post-hoc* (*p* values):

| **PC1** | | NOVX | DM-NOVX | OVX | DM-OVX |
| --- | --- | --- | --- | --- | --- |
| NOVX | |  | <0.01 | 0.884 | <0.01 |
| DM-NOVX | | <0.01 |  | <0.001 | 0.427 |
| OVX | | 0.884 | <0.001 |  | <0.01 |
| DM-OVX | | <0.01 | 0.427 | <0.01 |  |
|  |  |  |  |  |  |
| **PC2** | | NOVX | DM-NOVX | OVX | DM-OVX |
| NOVX | |  | 0.058 | 0.963 | 0.145 |
| DM-NOVX | | 0.058 |  | 0.064 | 0.615 |
| OVX | | 0.963 | 0.064 |  | 0.157 |
| DM-OVX | | 0.145 | 0.615 | 0.157 |  |

Significant results marked in red;
df—degrees of freedom, SS—sum of squares, MS—mean squares, F—F statistic, *p*—*p* value
